# Physio-biochemical and metabolomic responses of the woody plant Dalbergia odorifera to salinity and waterlogging

**DOI:** 10.1186/s12870-024-04721-5

**Published:** 2024-01-13

**Authors:** El- Hadji Malick Cisse, Bai-Hui Jiang, Li-Yan Yin, Ling-Feng Miao, Da-Dong Li, Jing-Jing Zhou, Fan Yang

**Affiliations:** 1 School of Ecological and Environmental Sciences, Hainan University, Haikou, 570228 China; 2 School of Life Sciences, Hainan University, Haikou, 570228 China; 3 Key Laboratory of Agro-Forestry Environmental Processes and Ecological Regulation of Hainan Province, Center for Eco-Environmental Restoration Engineering of Hainan Province, Haikou, 570228 China; 4 School of Plant Protection, Hainan University, Haikou, 570228 China

**Keywords:** Abiotic stresses, Adaptability, Antioxidant, Medicinal tree, Metabolomic, Phenylpropanoids

## Abstract

**Background:**

Trees have developed a broad spectrum of molecular mechanisms to counteract oxidative stress. Secondary metabolites via phenolic compounds emblematized the hidden bridge among plant kingdom, human health, and oxidative stress. Although studies have demonstrated that abiotic stresses can increase the production of medicinal compounds in plants, research comparing the efficiency of these stresses still needs to be explored. Thus, the present research paper provided an exhaustive comparative metabolomic study in *Dalbergia odorifera* under salinity (ST) and waterlogging (WL).

**Results:**

High ST reduced *D. odorifera*'s fresh biomass compared to WL. While WL only slightly affected leaf and vein size, ST had a significant negative impact. ST also caused more significant damage to water status and leaflet anatomy than WL. As a result, WL-treated seedlings exhibited better photosynthesis and an up-regulation of nonenzymatic pathways involved in scavenging reactive oxygen species. The metabolomic and physiological responses of *D*. *odorifera* under WL and salinity ST stress revealed an accumulation of secondary metabolites by the less aggressive stress (WL) to counterbalance the oxidative stress. Under WL, more metabolites were more regulated compared to ST. ST significantly altered the metabolite profile in *D. odorifera* leaflets, indicating its sensitivity to salinity. WL synthesized more metabolites involved in phenylpropanoid, flavone, flavonol, flavonoid, and isoflavonoid pathways than ST. Moreover, the down-regulation of L-phenylalanine correlated with increased p-coumarate, caffeate, and ferulate associated with better cell homeostasis and leaf anatomical indexes under WL.

**Conclusions:**

From a pharmacological and medicinal perspective, WL improved larger phenolics with therapeutic values compared to ST. Therefore, the data showed evidence of the crucial role of medical tree species’ adaptability on ROS detoxification under environmental stresses that led to a significant accumulation of secondary metabolites with therapeutic value.

**Supplementary Information:**

The online version contains supplementary material available at 10.1186/s12870-024-04721-5.

## Background

Investigation of medicinal species of the plant kingdom has been an essential activity of humankind since the pre-Christian era [1]. The crucial role of the growing conditions on plants related to synthesizing plant secondary metabolites (SMs) has been well-established. Indeed, soil water availability, temperature, light regime, or nutrient supply are deeply involved in plant secondary byproduct synthesis [2, 3]. Medicinal plants under water deficiency conditions showed considerably higher concentrations of natural products related to SMs than identical plants of the same species under ordinary environments. Those natural products include simple or complex phenols and numerous terpenes, alkaloids, flavonoids, or glucosinolates [4]. In the past several decades, stress induction has been used as a practical strategy to raise the accumulation of secondary metabolites. These metabolites are prominent under stress in phytochemistry and plant defense systems [5]. Moreover, a recent report pointed out that plants under harsh conditions show a potential alternative source for drug discovery [6]. This hypothesis is based on the fact that plants subjected to abiotic stresses generate significant organic compounds to protect several structures in plant organisms (cells and tissues). Various secondary metabolites contribute to stress resistance, responding to the mushrooming of reactive oxygen species (ROS), commonly known as oxidative stress [7].

Research has provided different indications that secondary metabolism in plants is related to oxidative stress [8]. Indeed, many SMs play the role of antioxidants in plant structures under environmental stresses. Naturally, a balance exists between the production of ROS and the synthesis of antioxidant molecules in plant systems [9, 10]. The most well-known reactive oxygen species are the superoxide radical anion (O_2_^●−^), hydroxyl radical (^●^OH), singlet oxygen (^1^O_2_), and hydrogen peroxide (H_2_O_2_) [11]. Plants have developed numeral mechanisms to shield themselves from oxidative damage caused by harsh environmental conditions [12–14]. The first component is the enzymatic machinery which is composed of various antioxidants such as ascorbate peroxidase (APX), superoxide dismutase (SOD), peroxidase (POD), glutathione peroxidase (GPx), catalase (CAT). Meanwhile, the second part involves non-enzymatic molecules such as ascorbic acid (ASA), reduced glutathione (GSH), carotenoids (CARs), and phenolic compounds (PHCs) [15, 16]. The antioxidant capacity of these phenolics is related to their ability to trap free radicals due to their appropriate structure (aromatic ring with –OH or –OCH_3_ substituent) [16–18]. The antioxidative systems described above still do not give the whole picture of ROS regulation during stress. Along with PHCs, a manifold of enzymes such as phenylalanine ammonia-lyase (PAL), plant lipoxygenase (LOX), polyphenol oxidase (PPO), or alternative oxidase (AOX) is involved in ROS induction and/or inhibition as well as in phenolic compounds biosynthesis. The enzyme PAL catalyzes the first step of the phenylpropanoid pathway that contributes to the synthesis of many SMs [19, 20]. In tandem with PAL, PPO affects the metabolism of phenolic compounds and their derivatives. Indeed, PPO induces the oxidation of phenolic compounds into highly reactive quinones. It might interact with peroxidase to promote ROS scavenging in plants under stress [21–23]. The enzyme LOX is involved in various physiological processes, including responses to environmental stresses [24], indeed, LOX is involved in forming singlet oxygen ^1^O_2_ [25]. Indeed, it has been shown that LOX and its products accumulate temporarily under diverse abiotic stresses [25, 26]. Waterlogging (WL) and salinity (ST) are well-known stresses that can massively provoke the accumulation of ROS in plants. Therefore, they can easily activate both enzymic and non-enzymic systems against oxidative stress.

ST and WL conditions are hostile environments for plant growth and development. There are two main components related to salinity: The osmotic component that reduces root water uptake and an ionic aspect that leads to ion toxicity, negatively affecting the photosynthetic apparatus [27, 28]. Meanwhile, soil waterlogged restraints the oxygen mobility between plants and their environment. The properties of the soil chemistry change during waterlogging, limiting the growth and distribution of certain species [29–31]. Several studies demonstrated that both stresses significantly increase medicinal plants' antioxidants and SMs [32–34]. It has been mentioned in [35] that research on medicinal plants is a new approach in the field that provides a broad range of possibilities for plant physiologist. The present study is unique because it emphasized from a plant physiologist's view the antioxidant functions of related therapeutic metabolites and compounds in trees under various environmental stresses depending on the species’ adaptability.

The present study highlights the metabolomic, anatomical, physiological, and biochemical responses of the medicinal woody plant *Dalbergia odorifera* (leaflets) under waterlogging and salt stress. *D. odorifera* belongs to the genus *Dalbergia*, family Fabaceae (Leguminosae). It is a medium-sized evergreen tree endemic to Hainan Island, South China [36]. In addition, *D. odorifera* has been regarded as a valuable plant for treating cardiovascular diseases, and previous reports indicated that it can prevent the occurrence of myocardial infarction. Several metabolites with medicinal value, such as Biochanin A (O-methylated isoflavone) and Genistein (C15H10O5) of *D. odorifera* were widely used to treat cardiovascular diseases, blood disorders or ischemia [37]. However, no study has focused on its metabolome profile under stress, and little is known about its molecular stress tolerance. A recent study has suggested that *D. odorifera* is an excellent tree to use in wetlands [38]. A recent article acknowledged how varying stress levels affect plant metabolism and performance. It highlights that low-stress levels can stimulate plant metabolism, which can be advantageous. However, high stress levels harm overall plant performance, limiting metabolic capacity and decreasing yield. It concludes that mild stress can benefit plants, and excessive stress negatively impacts their growth and productivity [39]. However, there is still a gap in understanding how the adaptability of plant species can increase metabolites with therapeutic value. The synthesis of by-products with therapeutic value in medicinal plants at early stages might depend on the specie’s adaptability and the type of abiotic stress. Thus, the present study aimed to decipher the metabolomic and biochemical profiles of *D. odorifera* leaflets under WL and ST. Most of the therapeutic substances are related to polyphenol biosynthesis. This comparative study aimed to decipher different metabolite pathways, such as phenylpropanoids, flavonoids, isoflavonoids, flavonol, and flavanone biosynthesis.

## Results

### Plant growth performance, water status, membrane permeability and anatomy of *D. odorifera* leaflet under stresses

**Fig. 1:**
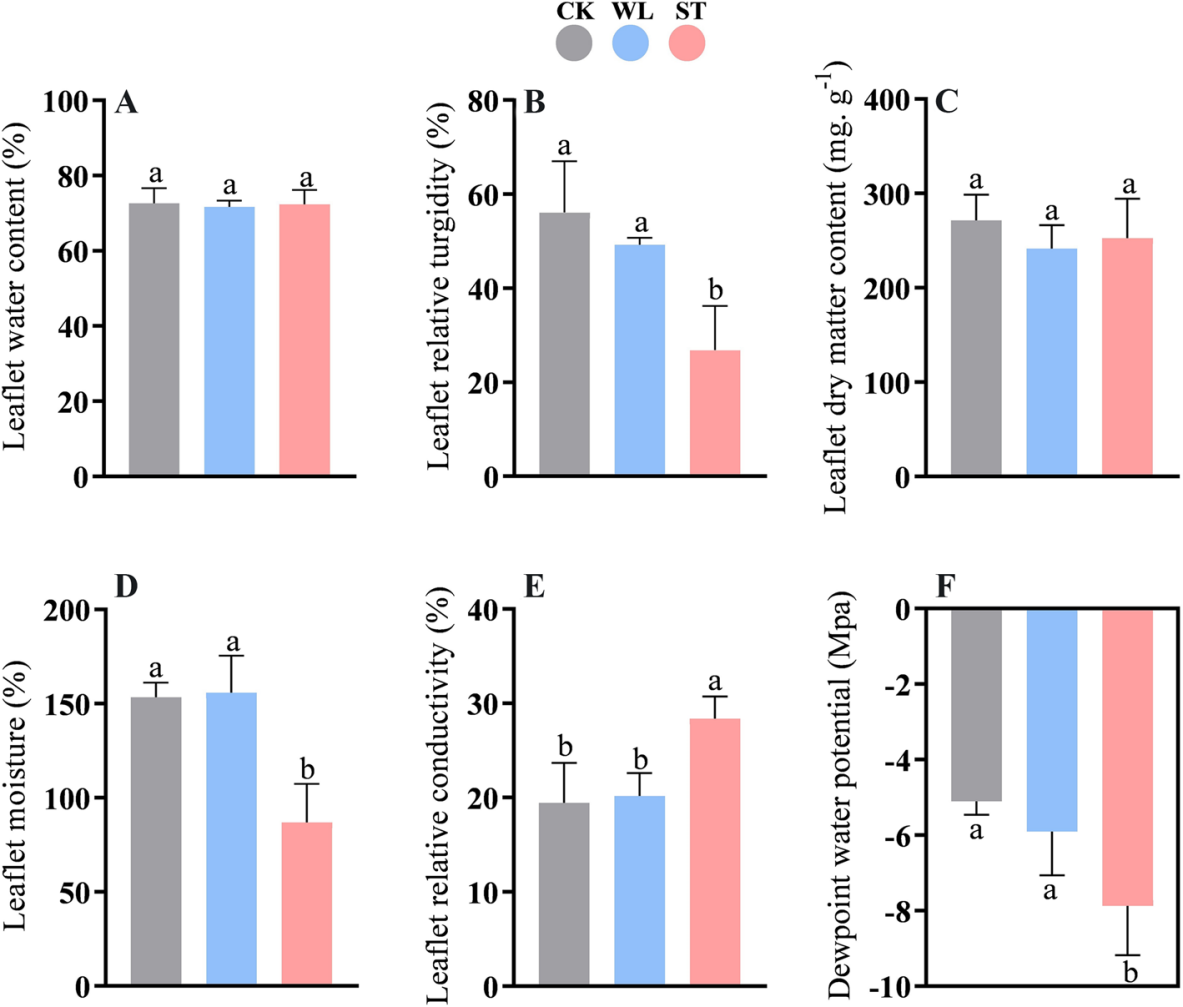
Leaflet water content (**A**), leaflet relative turgidity (**B**), leaflet dry matter (**C**), leaflet moisture (**D**), leaflet relative conductivity (**E**) and dew-point water potential (**F**) in Dalbergia odorifera leaflets under waterlogging and salinity. The bars on the top show standard error and different lowercases indicate significant difference among different treatments according to Tukey’s multiple comparison tests, respectively (*P* < 0.05). CK: control; WL; waterlogging; ST; salinity

The root and leaf biomass accumulation is presented in Table 1. ST (salinity)-treated seedlings showed a significant decrease in shoot-fresh weight compared to WL (waterlogging)-treated and control groups (CK). However, the difference in shoot-dry weight was non-significant in all treatments. The fresh and dry root weights were decreased in WL and ST treatment compared to CK. The present research explored the quality and physiological properties of *D. odorifera* leaflets via different indexes related to water loss and potential. The results about the water status of *D. odorifera* under ST and WL are shown in Fig. 1, where changes in leaflet water content (LWC), relative turgidity (LRT), and moisture (LM) are expressed in percent. It is apparent that the change in LRT under salinity was significant (*p* ≤ 0.05) compared to those in waterlogging-stressed seedlings (WL-stressed) (Fig. 1B). However, LWC showed statistically no difference in *D. odorifera* leaflet under different treatments. The values of dry matter (LDM) content and relative conductivity (LRC) in the leaflets of *D. odorifera* under ST and WL compared to the control group showed the same pattern as those in LWC (Fig. 1C, E). Meanwhile, the dew-point water potential (DPW) and LM followed the pattern of LTR. Overall, the significant decrease of LRT, DWP, and LM by salinity is critical for the water status and membrane permeability of the *D. odorifera* leaflet.

**Table 1:** Plant growth performance and leaflet anatomy indexes measurements of *D. odorifera* seedlings under severe salinity and waterlogging

Indexes	Treatments		
	**Control**	**Waterlogging**	**Salinity**
**Shoot fresh weight** (g)	45.1 ± 1.1 a	42.4 ± 1.6 a	38.2 ± 2.3 b
**Shoot dry weight** (g)	15.9 ± 0.5 a	15.2 ± 0.4 a	15.1 ± 0.9 a
**Root fresh weight** (g)	27.3 ± 1.4 a	22.5 ± 1.5 b	22.8 ± 0.6 b
**Root dry weight** (g)	8.9 ± 0.3 a	8.1 ± 0.3 b	8.1 ± 0.5 b
**Vascular bundle diameter** (μm)	38.5 ± 2.2 a	38.9 ± 1.3 a	32.5 ± 1.5 b
**Vein length** (μm)	168.9 ± 1.7 a	164.0 ± 1.3 a	148.9 ± 4.1 b
**Cross-section length** (μm)	122.8 ± 4.3 a	106.9 ± 3.8 b	107.6 ± 1.1 b
**Edge length** (μm)	136.8 ± 6.3 a	114.8 ± 1.1 b	101.4 ± 1.8 c

data are expressed as mean ± standard deviation, different lowercase letters indicate the significant difference after multiple comparisons test between different treatments at *P* < 0.05

**Fig. 2:**
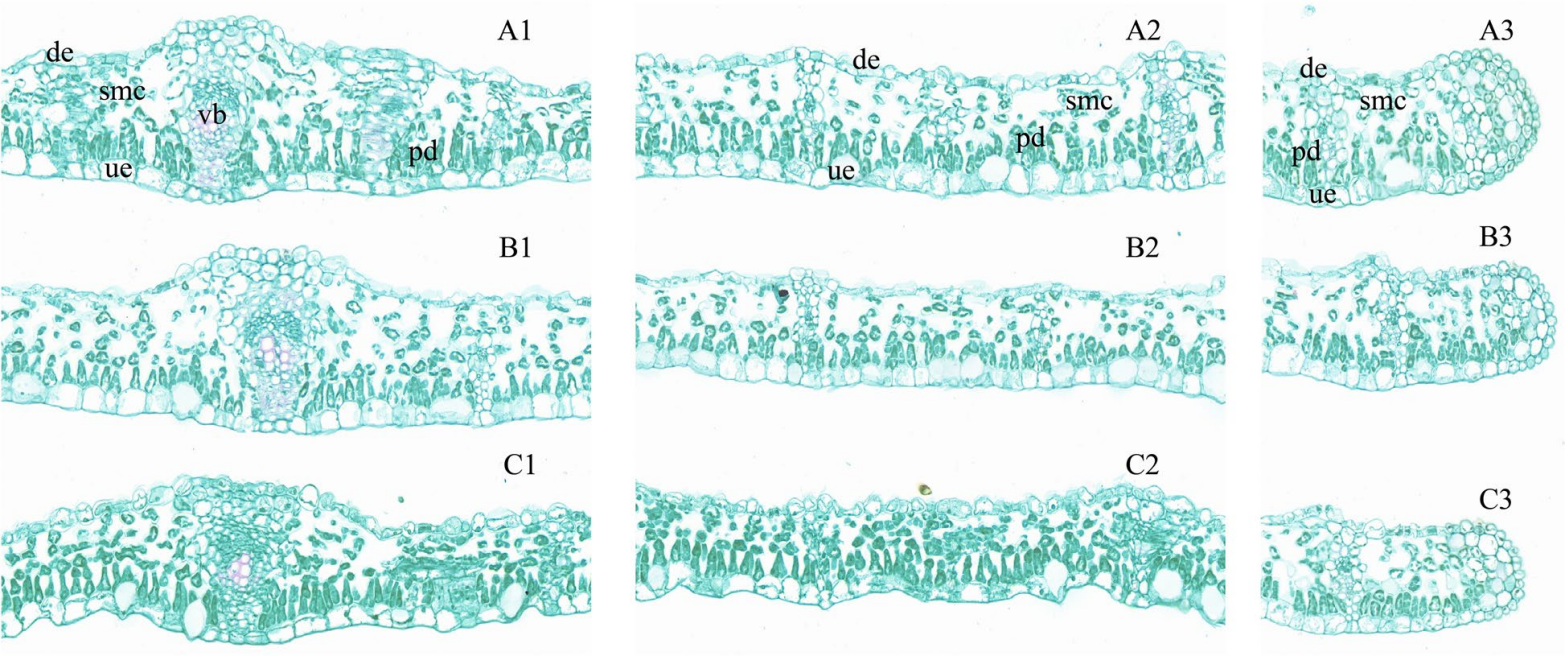
Anatomical changes of the leaflets of Dalbergia odorifera exposed to waterlogging and salinity with the control (A1-A3), WL (B1-B3) and ST (C1-C3) treatment. Row 1 (A1, B1 and C1) represents the vein of a leaflet, row 2 (A2, B2 and C2) the cross-section of a leaflet and row 3 (A3, B3 and C3) the edge of a leaflet. All images were taken at a set of 20 × zoom level with CaseViewer and scale bars = 50 μm. The letters within the images are: de, down-epidermis; pd, palisade mesophyll cell; smc, spongy mesophyll cell; ue, upper-epidermis; vb, vascular bundle

The anatomy indexes of *D. odorifera* leaflets under waterlogging or salinity compared to the control group are shown in Table 1 and Fig. 2. The leaflets of *D. odorifera* under different treatments were typically bifacial and composed of the upper (adaxial) and lower (abaxial) epidermis (single layers), spongy, and palisade tissue (mesophyll) (Fig. 2). The cells which composed the epidermis showed rectangle or oblong form. The lower epidermis was thinner than the upper epidermis. The epidermis cells were organized closely without intercellular space. Four samples were selected for each treatment to measure the average length between the upper and lower epidermis in the veins (Fig. 2 A1, B1, and C1), cross-sections (Fig. 2 A2, B2, and C2) and, the edges (Fig. 2 A3, B3, and C3) of *D. odorifera* leaflets. The leaflet anatomy of *D. odorifera* was compared between seedlings treated with salinity and waterlogging and a control group. The study found that the stress-treated seedlings significantly reduced the length of the edges and cross-section areas compared to the control group. This reduction had a significant impact on the size of the leaflets, as indicated in Table 1. The diminution of the size of *D. odorifera* leaflets, which also affects the leaflet thickness, can be explained by the overall reduction in spongy and palisade mesophyll cell size and areas. However, the transverse section of *D. odorifera* leaflets from waterlogging-treated seedlings did not differ notably from that of the control plants in the length of the vein and vascular bundle diameter. Indeed, salinity affected *D. odorifera*’ leaflet morphology and anatomy more than waterlogging did in comparison with the control group.

### Photosynthesis and oxidative stress in *D. odorifera* leaflet under stresses

The leaflets of the salt- and waterlogging-treated plants showed a significant (*p* ≤ 0.05) decrease in the carotenoid and total chlorophyll contents compared to the control group (Table 2). Under salinity and waterlogging, the leaflet photosynthetic rate (P_N_) decreased significantly compared to the control group. The P_N_ values in salt-treated seedlings were strongly lower than that in WL-treated plants. Additionally, the variation of the stomatal conductance (Gs) and transpiration rate (Trr) were significantly decreased by both stresses compared to the control group (Table 2). The difference in Trr and Gs values between salt-treated plants and WL-treated seedlings was not statistically significant. Furthermore, there was not a significant difference in water use efficiency (Wue) between the seedlings exposed to stress and the control group, nevertheless, the highest average value of Wue was found in WL-stressed seedlings.

**Table 2:** Total of chlorophyll (T-Chlo), carotenoid, photosynthetic rate (P_N_), Transpiration rate (Trr), stomatal conductance (Gs), and water use efficiency (Wue) variations of *D. odorifera* leaflet grown under salinity and waterlogging

Treatments	Photosynthetic indexes					
	**T-Chlo** µg g^−1^(f.m.)	**Carotenoid** µg g^−1^(f.m.)	**P**_**N**_ μmol m^−2^ s^−1^	**Trr** mmol m^−2^ s^−1^	**Gs** mol m^−2^ s^−1^	**Wue** µmol mmol^−1^
**Control**	1300.7 ± 075.7 a	246.5 ± 11.2 a	7.0 ± 0.3 a	4.1 ± 0.22 a	0.12 ± 0.011 a	1.68 ± 0.1 a
**Waterlogging**	986.6 ± 105.1 b	201.3 ± 27.2 a	3.9 ± 0.6 b	2.0 ± 0.80 b	0.05 ± 0.023 b	2.11 ± 0.7 a
**Salinity**	742.5 ± 120.1 b	136.9 ± 30.5 b	2.8 ± 0.1 c	1.7 ± 0.04 b	0.04 ± 0.004 b	1.5 ± 0.3 a

data are expressed as mean ± standard deviation, different lowercase letters indicate the significant difference after multiple comparisons test between different treatments at *P* < 0.05

The oxidative stress stimulated by ROS accumulation in *D. odorifera* was strongly more marked in seedlings exposed to salinity than those under waterlogging (Table 3). The increase of H_2_O_2_ in ST-treated plants was very slightly compared to WL-treated seedlings (11.28%). Meanwhile, ST has enhanced ^●^OH (85.57%) and O_2_^●−^ (36.61%) significantly compared to those in WL-treated seedlings. The formation of MDA which ROS can induce followed the same pattern as ^●^OH and O_2_^●−^. The lipid peroxidation between the control group and WL-treated seedlings was statistically similar. The MDA content in ST-treated seedlings was drastically raised (p ≤ 0.05) compared to the control group (51.97%) and WL-treated seedlings (25.62%).

**Table 3:** Variations of the oxidative indexes including the reactive oxygen species (H_2_O_2_, ^●^OH and O_2_^●−^), malondialdehyde, antioxidant molecules (AsA, GSH and AOX), and antioxidant enzymes (POD, SOD, CAT and GPX)

Indexes	Treatments		
	**Control**	**Waterlogging**	**Salinity**
**H**_**2**_**O**_**2**_ (μmol/g•Fw)	58.07 ± 7.73 b	143.39 ± 4.88 a	159.57 ± 11.68 a
**●OH scavenging capacity** (A•1000/g•Fw)	71.19 ± 16.6 c	737.79 ± 43.3 a	397.78 ± 19.07 b
**O**_**2**_^**.−**^** Production rate** (nmol/g•Fw/min)	40.45 ± 3.6 c	70.40 ± 2.8 a	50.76 ± 6.4 b
**MDA** (µmol/g•Fw)	26.71 ± 2.8 c	39.09 ± 2.5 a	31.85 ± 2.8 b
**AsA** (nmol/g•Fw)	244.88 ± 29.7 a	222.47 ± 3.9 a	133.84 ± 14.5 b
**GSH** (µg/g•Fw)	147.57 ± 10.1 b	287.10 ± 15.7 a	284.51 ± 12.1 a
**AOX** (ng/mg•Fw)	0.023 ± 0.002 a	0.025 ± 0.002 a	0.021 ± 0.002 a
**POD** (UA /mg•FW/min)	4.375 ± 1.3 b	9.68 ± 1.2 a	5.75 ± 0.8 b
**SOD** (UA/mg•FW)	0.046 ± 0.01 c	0.21 ± 0.01 a	0.12 ± 0.02 b
**CAT** (UA/mg•FW/min)	0.21 ± 0.04 b	0.55 ± 0.03 a	0.17 ± 0.02 b
**GPX** (UA /mg•FW/min)	2.66 ± 0.8 a	2.57 ± 0.08 a	2.68 ± 0.09 a

data are expressed as mean ± standard deviation, different lowercase letters indicate the significant difference after multiple comparisons test between different treatments at *P* < 0.05

The enzymes such as SOD, CAT, and POD which are not directly related to medicinal compounds in comparison with the phenolics were more dynamic in ST-treated seedlings compared to those exposed to WL. Indeed, the activities of POD, SOD, and CAT were strongly higher (*p* ≤ 0.05) in plants under ST than that in WL-treated seedlings and the control group (Table 3). The glutathione peroxidase activity didn’t show significant changes in *D*. *odorifera* plants under stress (Table 3). The AOX protein seemed to not involve in stress responses at early stages in *D. odorifera* under ST or WL. The accumulation of AOX was statistically similar between the control group and ST- and WL-treated seedlings (Table 3). Moreover, the accumulation of antioxidant molecules that are not phenolic compounds showed an increase with GSH by ST and WL treatments compared to AsA which showed a decrease by WL treatment compared to the control group (Table 3).

### Phenolic compounds and related enzymes activities in *D. odorifera* leaflet under stresses

**Fig. 3:**
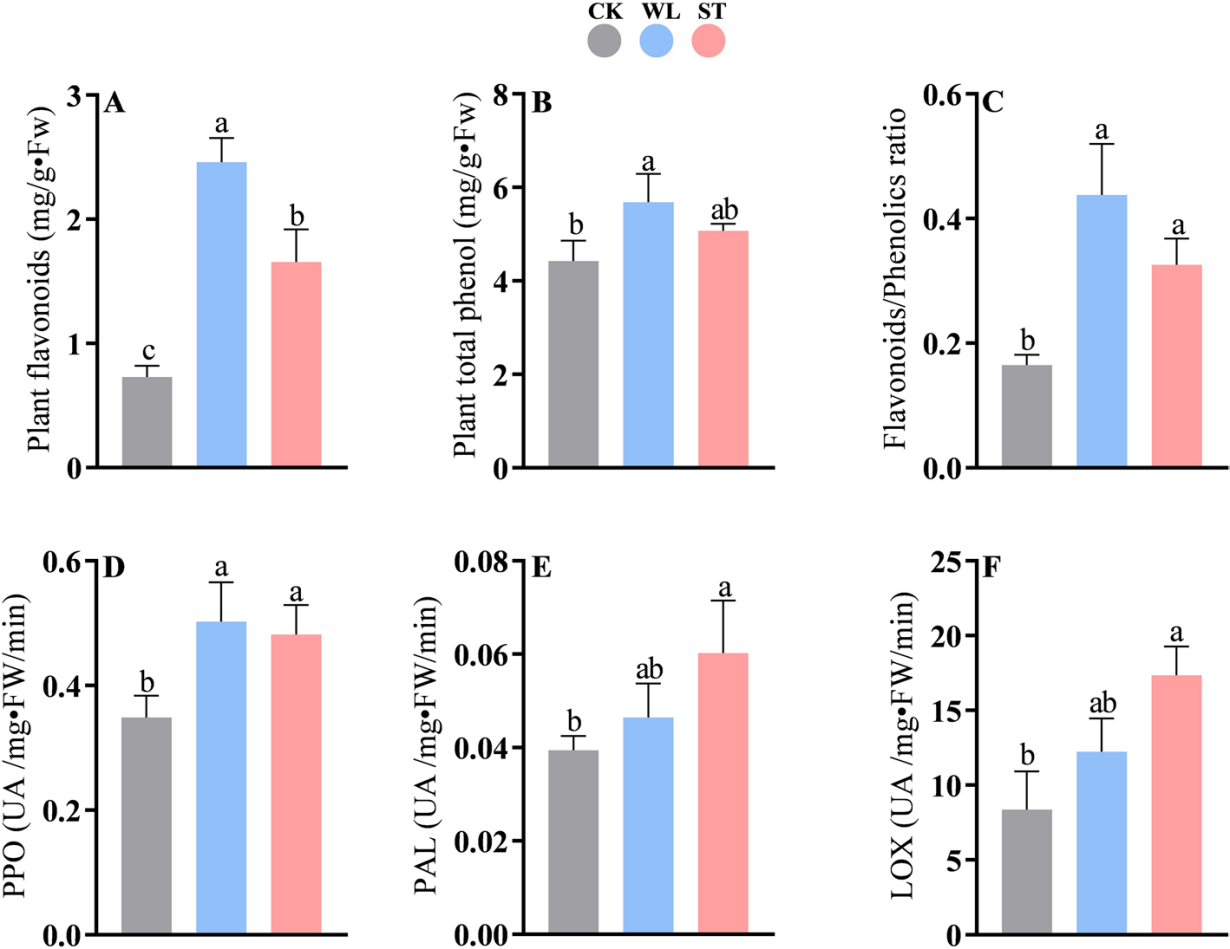
Plant flavonoids (**A**) and total of phenol (**B**) contents, ration between flavonoids and phenolics (**C**), polyphenol oxidase (PPO, **D**), phenylalanine ammonia-lyase (PAL, **E**) and plant lipoxygenase (LOX, F) activities in Dalbergia odorifera leaflets under waterlogging and salinity. The bars on the top show standard error and different lowercases indicate significant difference among different treatments according to Tukey’s multiple comparison tests, respectively (*P* < 0.05). CK: control; WL; waterlogging; ST; salinity

Phenolic compounds and flavonoids are well-known antioxidants with redox properties that participated in plant defense under abiotic stresses. The results for the total phenolic compounds and flavonoid content and the ratio of flavonoids/phenolics in *D. odorifera* under ST and WL are shown in Fig. 3. The data has shown that *D. odorifera* under waterlogging represented the richest source of phenolics and flavonoids compared to salinity (Fig. 3A-C). However, the increase in the total phenolics was not statistically significant between ST-treated and WL-treated seedlings. Both stresses strongly enhanced the plant flavonoid content compared to CK. Meanwhile, only WL significantly increased the phenolics compared to the control. *D. odorifera* under ST (122.91%) and WL (140.62%) showed a significant ratio of flavonoid/phenolics compared to the control. Thus *D. odorifera* under waterlogging and salinity represented a massive source of flavonoids.

ST and WL considerably raised the polyphenol oxidase (PPO) in D. odorifera seedlings. Between ST-treated and WL-treated seedlings, the PPO activity was similar (Fig. 3D). The phenylalanine ammonia-lyase (PAL) and plant lipoxygenase (LOX) follow the same pattern as PPO regarding their activities in ST-treated seedlings compared to the control group (Fig. 3D-E). The PAL and LOX activities slightly increased (non-significant) by WL compared to CK.

### Metabolomic analysis of *D. odorifera* leaflet under stresses

**Fig. 4:**
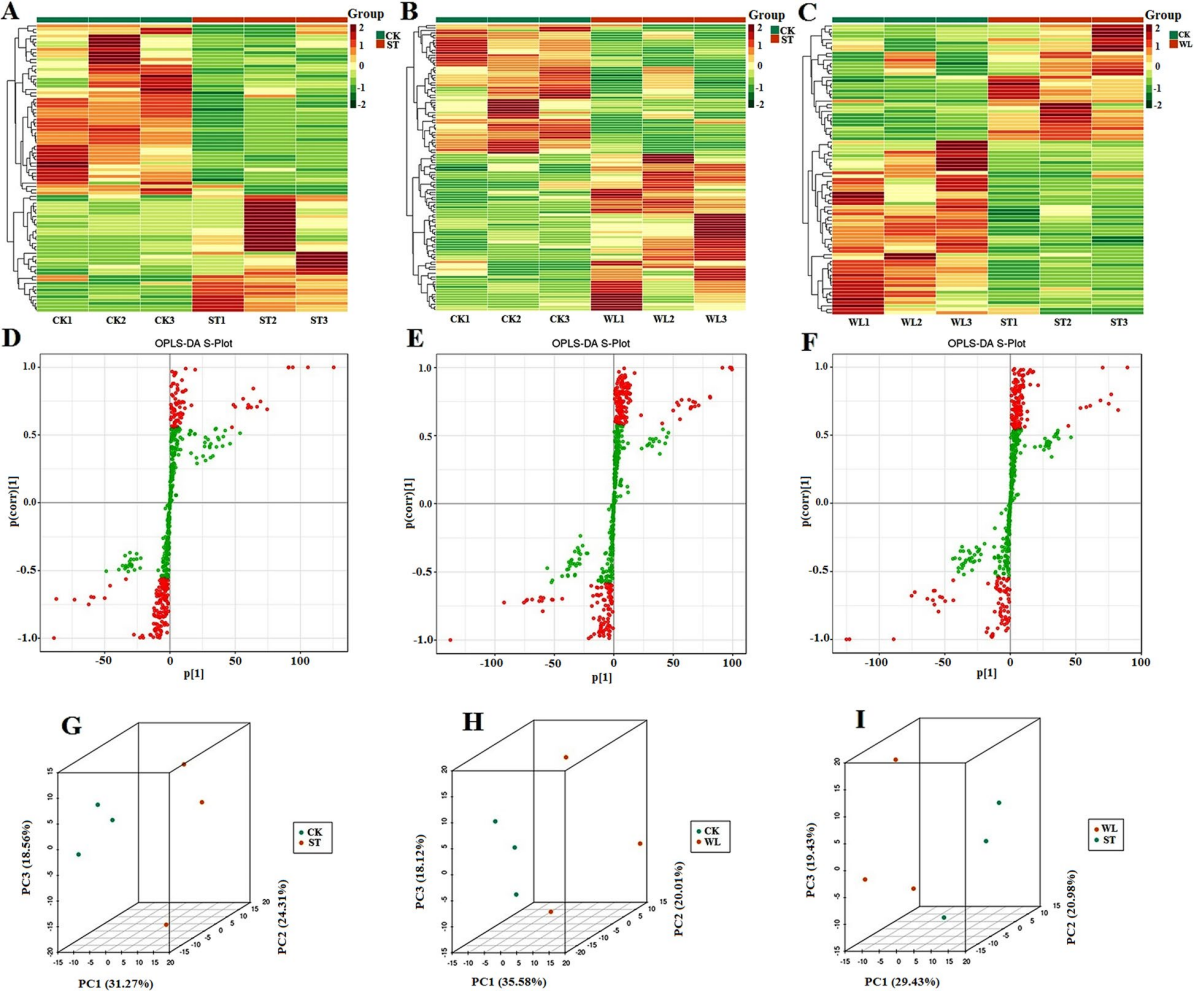
Samples clustering heatmap (**A**-**C**), OPLS-DA S-plot (red indicates metabolites with VIP ≥ 1, and green indicates metabolites with VIP < 1) (**D**-**F**), principal component analysis (PCA) grouped 3D plot (**G**-**I**) of the metabolome in D. odorifera leaflets under salinity and waterlogging. CK: control; WL; waterlogging; ST; salinity

Characterizing the metabolomic profile in stress-induced medicinal compounds will significantly add to our knowledge of how abiotic stress can lead to enhanced better production of therapeutic substances in medicinal plants, compared to other conditions. The increasing availability of data describing the enhancement of secondary metabolites by abiotic stress has helped us better understand the diverse functions of several metabolites, mainly phenolic compounds, in the plant's stress response.. Since these metabolites can play a crucial role in human health, this section will focus on deciphering the differences between stress-induced compounds under ST and WL conditions. To understand the difference in metabolomic analysis of *D. odorifera* leaflet under waterlogging versus salinity, the present section focused particularly on the comparison of WL-metabolomic data versus ST. Moreover, the present study aimed to perform the multivariate statistical analysis separately in CK vs ST, CK vs WL and ST vs WL to highlight the significance of difference for each group. Cluster analysis revealed significant regularity among replicated samples (Fig. 4A-C). OPLS-DA was performed to determine the significant differences in metabolomic profiles between WL-treated samples and ST and confirmed the PCA analysis (Fig. 4D-F). Indeed, subjecting the metabolite data to PCA has divided the control, ST, and WL groups following the PC1 which possess the most significant contribution in explaining the association of multiple datasets (Fig. 4G-I). The results of the PCA reflected an obvious difference existing between metabolomic profiles within these 3 groups of treatments. Moreover, the data showed that the DMs patterns were remarkably opposite between WL and ST groups. The results from the multivariate statistical analysis validated that the separation of the metabolomic profiles between ST and WL samples was stable and meaningful.

**Fig. 5:**
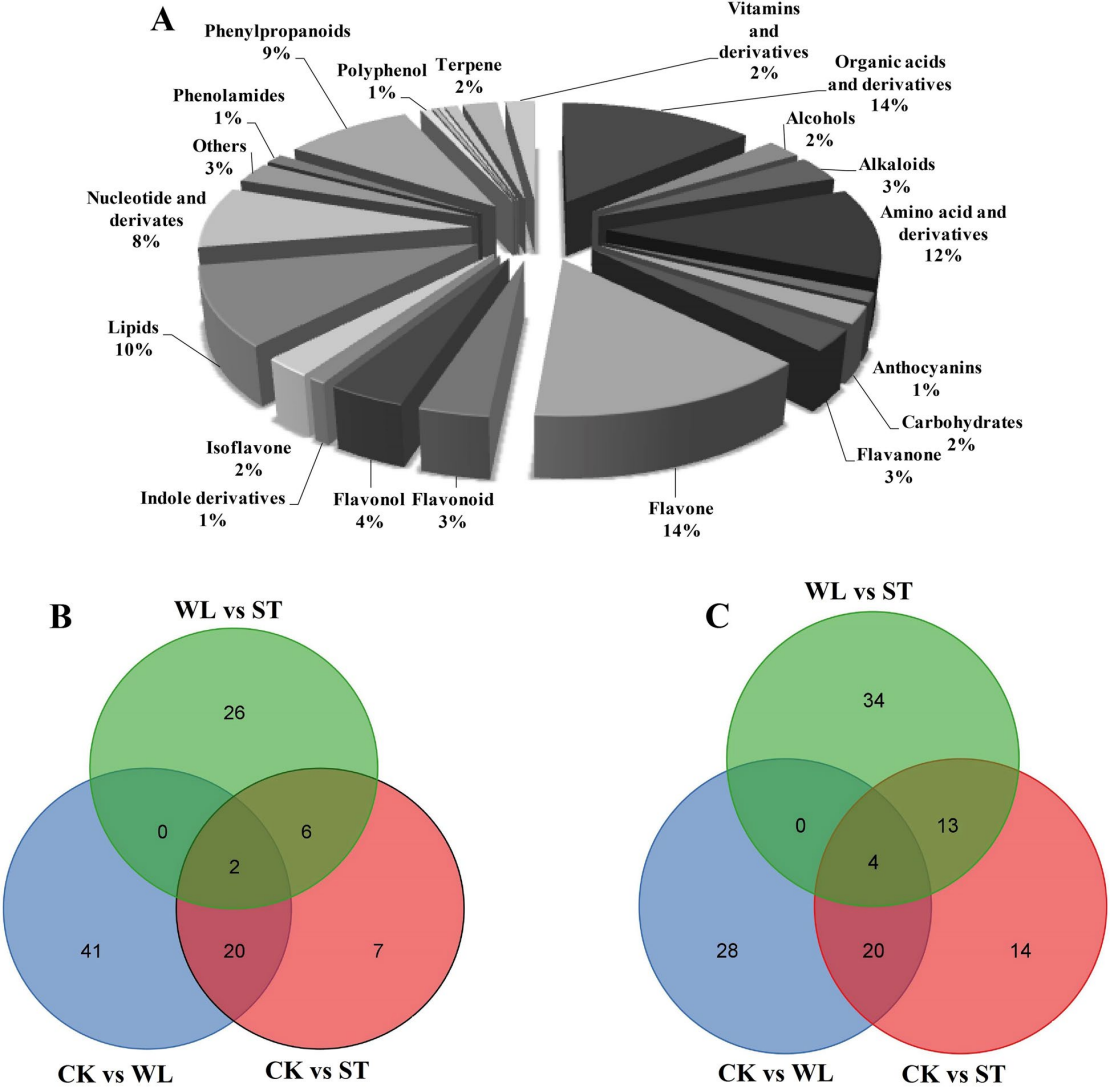
Different metabolites detected in Dalbergia odorifera leaflets under control (CK), waterlogging (WL) and salinity (ST) (**A**). Venn diagrams of the number of up-regulated (**B**) and down-regulated (**C**) differential metabolites in WL versus ST, CK vs WL and CK vs ST

The metabolomic analysis based on the KEGG database, MWDB, and MRM revealed 572 metabolites. The metabolites detected were composed of different classes including organic acids and derivatives (79), alcohols (14), alkaloids (19), amino acid and derivatives (66), anthocyanins (7), carbohydrates (13), flavanone (16), flavone (81), flavonoid (20), flavonol (22), indole derivatives (5), isoflavone (14), lipids (58), nucleotide and derivates (45), others (15), Phenolamides (7), phenylpropanoids (52), polyphenol (4), proanthocyanidins (2), quinones (2), sterides (5), terpene (14) and, vitamins and derivatives (12). Figure 5A displayed a clear dominance of the phenolics and their related metabolites detected in the *D. odorifera* leaflet under different treatments. Among the 572 differentially metabolites (DMs) identified, the expression levels of 20 common metabolites between *D. odorifera* ST and WL-treated leaflets were up-regulated or down-regulated compared to CT (Figs. 5B-C). Table 4 showed the numbers of DMs between CK vs ST, CK vs WL also WL vs ST. The highest number of DMs was found in CK vs WL group; also the related phenolics DMs followed the same pattern.

**Table 4:** Comparable analysis on the differential metabolites numbers in the leaflets of *D. odorifera* under waterlogging (WL) and salinity (ST) compared to control (CK) following selected group name class

Class	ST vs CK		WL vs CK		WL vs ST	
	**up**	**down**	**up**	**down**	**up**	**down**
**Flavone**	13	3	16	4	5	3
**Flavonol**	5	1	2	2	1	1
**Phenolamides**	1	–	–	–	–	1
**Flavonoid**	1	2	1	4	–	3
**Alkaloids**	–	1	0	1	2	2
**Phenylpropanoids**	–	5	3	12	2	5
**Isoflavone**	–	5	–	5	–	–
**Flavanone**	–	–	–	3	–	–
**Sterides**	–	–	–	1	–	1
**Anthocyanins**	–	1	–	–	–	1
**Organic acids and derivatives**	12	2	8	7	2	14
**Amino acid and derivatives**	8	4	14	6	13	5
**Nucleotide and derivates**	5	1	7	2	4	2
**Others**	6	10	1	16	5	13
**Total**	35	51	63	52	51	34
**All sig diff**	86		115		85	

Metabolites that exhibited a fold change greater than or equal to 2, a fold change less than or equal to 0.5, and a Variable Importance in Projection (VIP) score of 1 or higher were chosen. When comparing samples from WL and ST, it was observed that metabolites filtered based on these criteria demonstrated notable differences.

The differential metabolite KEGG functional annotation and enrichment analysis between WL and ST have been shown in Suppl. Figure 2. The differential metabolites (including CK vs ST/WL) were primarily active in metabolic pathways and the creation of secondary metabolites. These include various flavonoids (like Isoflavonoids, flavones, and flavonols) and phenol and phenolic biosynthesis compounds. Under WL the results revealed that 40 metabolites in *D. odorifera* leaflets were involved in secondary metabolites biosynthesis and 24 were involved (up-regulated) under ST (Suppl. Figures 3–4) compared to control. Moreover, the flavone and flavonol, flavonoids, and phenylpropanoids biosynthesis pathways followed the same pattern as the secondary metabolites pathway. Under waterlogging, there were more metabolites involved in these pathways compared to salinity in *D. odorifera* leaflets. However, a comparison between WL and ST in Suppl. Figure 2 showed that there are a number of metabolites involved in various metabolism that were up-regulated under salinity compared to WL treatment. The statistics of the differential metabolite KEGG enrichment maps in *D. odorifera* leaflets under CK vs ST (Suppl. Figure 5A), CK vs WL (Suppl. Figure 5B) and WL vs ST (Suppl. Figure 5C) showed that WL-treated seedlings accumulate significantly more compounds related to secondary metabolites sub-group or metabolites involved in phenolic compounds biosynthesis.

## Discussions

The mechanisms by which abiotic stresses affect medicinal woody and their related therapeutic compounds are mostly unknown. Here is a specific case in a woody plant where it is studied and evaluated if the sensitivity to abiotic stress (salinity; ST) can induce more chemical compounds related to medicinal molecules compared to its tolerance to another different environmental stress (waterlogging; WL) at early stages. The results demonstrated that the type of stress and the adaptability of medicinal woody species improve differently the biosynthesis of secondary metabolites, phenolics compounds, and leave qualities. Indeed, the preliminary study has shown clearly that *D. odorifera* showed significant adaptability to WL compared to ST (Suppl. Figure 6). After one month of treatment, the survival rate of *D. odorifera* seedlings was 53% under ST (200 mM) and 100% under WL (Suppl. Table 1). And as showed the Suppl. Figure 7*D. odorifera* leaflets phenotype was not affect by necrosis or chlorosis under saline conditions at day 6 (early stages of stress).

### Waterlogging and salinity affected the morpho-physiology of *D. odorifera* leaflets without causing necrosis at the early stages

Abiotic stresses such as waterlogging or salinity affect leaf biomass accumulation in plants. Plants exposed to high salinity can experience several damages, including reduced shoot biomass and water content [17]. At early stages (6 days), *D. odorifera* showed similar shoot dry weight accumulation under ST conditions compared to control (CK). Meanwhile, the shoot fresh weight was significantly lower in ST-treated seedlings. Indeed, salinity affects leaf biomass by reducing the amount of water plants can absorb from the soil, ultimately decreasing leaf biomass. However, [40] showed experimentally how salinity affects leaf dry biomass following the stress level and the duration of salinity. Indeed, ST can increase or sustain the leaf dry biomass by accumulating solutes or metabolites, such as inorganic ions, sugars, amino acids, or phenylpropanoids in the plant cells. These compounds help to maintain the osmotic balance during harsh conditions. Indeed the effects of salinity on leaf fresh or dry mass accumulation will depend on various factors, including the plant species, the level of ST, and the duration of exposure to salt [41, 42]. High ST generally decreases biomass accumulation and productivity, including leaf dry mass accumulation reductions. The results suggested that at early stages of stress, *D. odorifera* can maintain its leaflet water content with a high production of phenolics compounds which provides a significant perspective of the use of salt stress-induced medicinal compounds in *D. odorifera*.

Plant leaves are highly complex and unstable physico-biological system structures in form, longevity, anatomical architecture, and capacity for photosynthetic gas exchange [43, 44]. The water potential (DWP), leaflet relative turgidity (LRT), leaflet dry matter (LDM), leaflet moisture (LM), leaflet relative conductivity (LRC), and the leaflet water content (LWC) were evaluated to highlight the leaf water loss and hydraulic conductance in *D. odorifera* leaflet. Indeed, leaf water conductance and potential greatly influence water movement throughout the plant [45]. The water potential, leaflet moisture, and relative turgidity were significantly affected by salinity compared to waterlogging at the early stages. WL and ST, as one of the most threatened environmental stresses, can drastically reduce LWC and increase LRC that provoke turgor loss, closure of stomata, inhibition of cell enlargement, and reduces plant growth that affects leaflet photosynthesis [46–48]. This research observed no significant difference in leaf water status between WL-treated seedlings and control. Based on the model explained by [44], it is evident that the significant decrease in the leaflet vascular bundle diameter and the vein length are partly responsible for the negative effect of ST on the leaflet water indexes, which are related to their leaflet hydraulic conductance. The results of Buckley were based on the variation of the vein size and leaf area across species. In the current study, the variation in the size of the leaf and vein area is caused by abiotic stresses in the same tree species, in which harsh conditions caused a significant (ST) or slight (WL) reduction of the leaf and vein area. Indeed, a relationship exists between the thickness of mesophyll tissue, the proportion of spongy to palisade mesophyll tissue thickness, and the rate of water movement through the leaf (hydraulic leaf conductance). The leaflet vein area was significantly similar to those in the control, which could explain why the leaflets in WL-treated seedlings showed no significant reduction in LWC and DWP, or increase in LRC compared to the control. In fact, larger veins are more efficient for water transport, which would result in lower water conductivity found in the control and WL group [49]. Furthermore, leaf anatomy, water status, and photosynthetic efficiency are correlated from each other. The transpiration rate (Trr) and net photosynthetic rate (PN)were decreased by WL and ST treatments as expected. Indeed plant under stress tends to reduce photosynthetic activity as a responsive defence mechanism to use more efficiently its energy. The responsive mechanisms of plants under environmental stresses require massive consumption of energy. Various plant vital processes such as photosynthesis or cell growth are affected to conserve energy in plants under stressful conditions [50]. The explanation of the decrease in photosynthetic pigments, P_N_ and Trr was more related to the variation of the stomatal conductance (Gs) in the present study. Both WL and ST were able to decrease the Gs at early stages, and even though the control group and WL-treated seedlings showed the same water status, vein length and vascular bundle diameter, the photosynthesis was significantly reduced by WL. Meanwhile, there was a positive correlation among the decrease in vein area, mesophyll tissue, Gs, and photosynthesis in the seedlings exposed to salinity.

### Different pathways to deal with oxidative stress in *D. odorifera* leaflets following the sensitivity to ST or tolerance to WL

The present study aimed to evaluate ROS levels in *D. odorifera* leaflets at early stages under WL and ST due to their ability to damage cell redox-homeostasis, affecting the leaflet water status and metabolism. Owing to the fact that ROS has a multitude of cellular functions such as the regulation of plant cell development and differentiation, redox-homeostasis, stress signaling, interactions with other organisms, systemic responses, and plant cell death [51].

Plants under abiotic stress use mainly an antioxidant system composed of enzymatic and non-enzymatic components to prevent ROS over-accumulation in plant cells and lessen its harmful effects [16, 52–54]. The oxidative damages caused by ROS were more significant in ST-treated seedlings compared to those in WT-treated plants. To face the over-accumulation of ROS, *D. odorifera* leaflets showed higher enzymic activities under ST compared to WL. In fact, the traditional enzymatic antioxidant system including SOD, POD, and CAT was clearly the pathway chosen by *D. odorifera* leaflets to deal with ROS over-production under salinity. However, GPX activity was statistically similar in *D. odorifera* leaflets under different treatments, which suggests a significant role in ROS scavenging under normal conditions. The role of many enzymic antioxidants has been extensively studied, for instance, as [52] explained so well that SOD releases superoxide radicals by dismutation to form hydrogen peroxide; CAT decomposes hydrogen peroxide to form H_2_O and O_2_ and POD detoxify H_2_O_2_ via different substrates such as guaiacol. However, the difference in PAL, PPO, and LOX activities was not significant between WL-treated and ST-treated seedlings. Indeed, PAL and PPO enzymes are indirectly involved in ROS regulation and detoxification via phenolics biosynthesis pathways. The increase of PAL by ST and WL in the present study is more related to its role linked with the regulation phenylpropanoid pathways as described [19] in a view of the positive correlation between PAL activity, plant total phenol, and flavonoid under both stresses. Indeed, the biosynthesis of a wide range of phenylpropanoid-derived secondary products in plants such as flavonoids is triggered by the catalyzation of the non-oxidative deamination of phenylalanine to cinnamic acid by PAL. Meanwhile, PPO is an oxidoreductase enzyme that catalyzes the oxidation of monophenols and/or o-diphenols to highly reactive o-quinones, which can interact with oxygen to form reactive oxygen species [21, 22]. The LOX activity in *D. odorifera* leaflets was observed under both stresses, however the significance difference compared to the control group was found in ST-treated seedlings. The results showed a positive correlation between ROS accumulation and LOX, which support the findings of [55]. In the present study, the non-enzymic antioxidant system was composed of: ascorbic acid, (AsA), glutathione (GSH), phenolic compounds (alkaloids, flavonoids, etc.), and carotenoids. At this stage of stress, GSH was strongly involved in the oxidative response of *D. odorifera* leaflets compared to ASA and the carotenoids. Meanwhile, the phenolic compounds and particularly the flavonoids were clearly more involved in WT-responses compared to ST. The tolerance of *D. odorifera* seedlings to WL seems to induce more the lower-weight molecular antioxidant molecules against oxidative stress compared to the sensitivity of *D. odorifera* to ST which favoured the enzymic antioxidative pathway. Moreover, the phenolics compounds were more efficient in ROS scavenging compared to AsA under both stress. It has been demonstrated that flavonoids are able to surpass antioxidants like ascorbate in ROS scavenging because of their strong capacity to donate electrons or hydrogen atoms [56]. Furthermore, there is this idea that the AOX pathway is involved in photorespiration and antioxidative metabolism besides mitochondrial respiration. The hypothesis was brought out by [57] based on research work on *Arabidopsis* leaves [58]. Indeed, this report highlighted a possible involvement of the AOX pathway in ascorbate biosynthesis, following the fact that the over-expression of AOX1 was correlated to higher rates of ascorbate biosynthesis. The current results found in *D. odorifera* leaflets contribute to demonstrate that there is a positive correlation between the decrease/increase of AOX proteins and ascorbate production.

### The comprehensive metabolic analysis gives the edge to waterlogging in improving metabolites related to medicinal compounds

By using the data from metabolomic analyses, phenolics metabolisms have been identified as prominent drivers of stress responses in *D. odorifera* leaflets. Based on the KEGG database, MWDB, and MRM, there was a clear total of percentages showed in Fig. 5 that confirmed the omnipresence of secondary metabolites such as flavone, flavonol, flavonoid, phenylpropanoid, isoflavone or polyphenol involved in *D. odorifera* leaflets metabolisms compared to the metabolites related to organic acids biosynthesis, carbon metabolism or amino acids pathways under normal or stressed conditions. For instance, there were only 3 metabolites involved in carbon metabolism that differed from the control compared to WL or ST treatment. Indeed, in *Arabidopsis* plants the expression of multiple genes encoding enzymes of carbon metabolism and respiration was reduced by drought or heat [59]. The differential metabolites in the present research revealed that WL compared to CK up-regulated 63 and down-regulated 52 metabolites, meanwhile, in the ST-treated group it has been found that 35 metabolites were up-regulated and 51 down-regulated. Thus, suggesting that high ST alters the metabolites profile in *D. odorifera* leaflets which is significantly different from WL. It has been reported a decrease in the accumulation of metabolites in the leaves of *Medicago sativa* by ST [60]. The metabolism profile of a wild rice tolerant to high ST (200 mM) showed a total of 90 differential metabolites with 49 up-regulated and 41 down-regulated [61]. The lower number of the up-regulated metabolites in *D. odorifera* leaflet in ST-treated seedlings is obviously related to its sensitivity to high saline conditions.

**Fig. 6:**
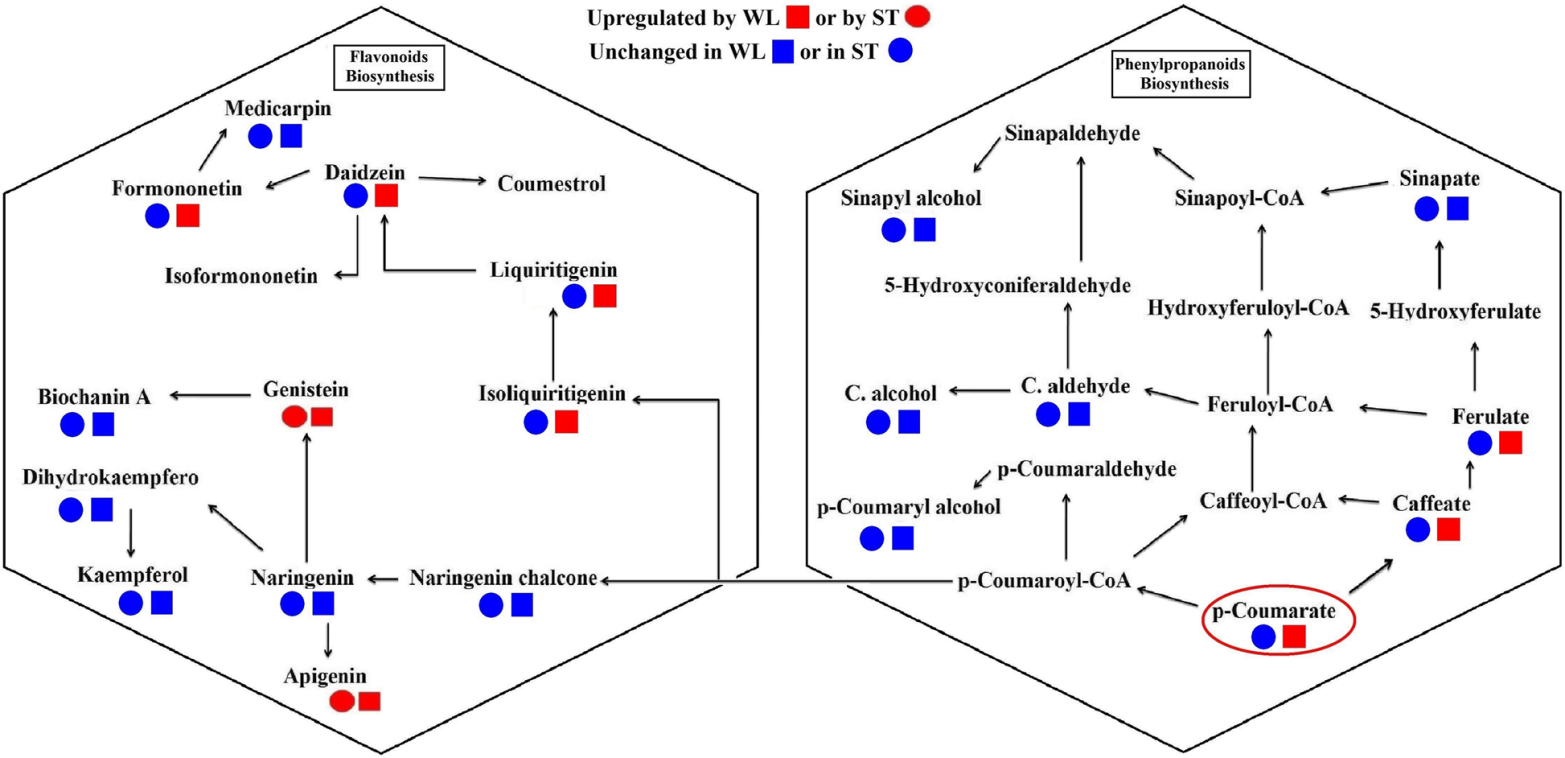
Overview of the secondary metabolites with therapeutic value profiling results in Dalbergia odorifera leaflets under waterlogging and salinity versus control

The focus on the secondary metabolites with a therapeutic value in the present study can be compared by metaphor as “the water that invariably returns to the source”. Indeed, it has been a thousand years since humans were interested in plant secondary metabolites for medicinal and pharmacological usages [62]. Nonetheless, the targeted metabolite analysis began approximately 200 years ago with the isolation of morphine from opium poppy (*Papaver somniferum*) [63]. KEGG/PATHWAY database [64] has been used to draw graphical diagrams to visualize the involvement of different phenolic metabolites with therapeutic values detected in *D. odorifera* leaflets in various metabolomic pathways under WL and ST as summarized in Fig. 6. Moreover, it has been performed a screening of this data to emphasize the involvement of different phenolic metabolites up-regulated in *D. odorifera* leaflets under stress. Most of the metabolites involved in phenylpropanoid, flavone and flavonol, flavonoid and, isoflavonoid pathway were unchanged under WL or ST (Suppl. Tables 2–6). The phenolics compounds up-regulated by WL outnumbered those in ST-treated seedlings. The explanation can be found in the level of metabolites such as L-phenylalanine or tryptophan. It appeared that both compounds which play a major role in the biosynthesis of phenolic compounds production showed a lower level in WL-treated seedlings. Indeed, the enzyme PAL catalyzes the reaction that transforms L-phenylalanine to p-coumarate which evolves in p-coumaroyl-CoA that represents the origin of the synthesis of myriad phenolics [65]. The low level of L-phenylalanine found in *D. odorifera* leaflets which showed a high accumulation of phenolics under WL is in agreement with the findings of [66] in tomato. Indeed, over-expression of a well-known gene (AtMYB12) in tomatoes involved in the phenylpropanoid pathway increased significantly the biosynthesis of flavonoids via a decrease of L-phenylalanine. It seems that the more L-phenylalanine is decreased, the more the phenolics are increased. Moreover, there were 3 well-known phenolics (p-coumarate, caffeate, and ferulate) that were up-regulated in phenolic coumarins pathway by WL and unchanged in ST treatment (Fig. 6).

These 3 metabolites are deeply involved in the biosynthesis of the cell wall polymer lignin as demonstrated [67]. Plant cell wall integrity plays a prominent role under stress in cell homeostasis [68], WL-treated seedlings showed a better water status and leaf anatomy indexes probably and partly due to the up-regulation of p-Coumarate, caffeate, ferulate and related metabolites. In *Arabidopsis*, it has been mentioned that p-coumaric acid, caffeic acid, and ferulic acid served as substrates for 4-coumarateCoA ligase which played a prominent role in cell lignifications [69]. Moreover, earlier work that used wounds to study the suberization of plant cells has shown that ferulates are the precursor of suberin [70]. The up-regulation of these three metabolites related to lignin and suberin can explain the shapes of cells and tissues in *D. odorifera* leaflets under WL and ST. Furthermore, for a pharmacological perspectives ferulates (up-regulated in WL-treated) and related polyphenols have been shown to inhibit cytotoxicity and oxidative stress in isolated rat hepatocytes [71]. The flavonoids pathway revealed 3 phenolic metabolites (genistein, apigenin, and pelargonidin) up-regulated by ST and 7 under WL (daidzein, liquiritigenin, butein, apigenin, genistein, formononetin, and isoliquiritigenin). A closer look at the flavonoids pathway consisted of an exploration of the isoflavonoid pathway and flavone and flavonol pathway exposed 4 more phenolic metabolites (rutin, glycitein, 2'-hydoxygenistein, and 6'-hydroxydaidzein) up-regulated in ST-treated seedlings and 5 more up-regulated by WL (vestitol, 2'-hydoxygenistein, maackiain, daidzein and, rutin). Most of the metabolites mentioned above have been reported to possess anticancer, antioxidative, or anti-inflammatory properties. Some of these metabolites showed multiple functions in plants and human health, for instance, it has been reported that apigenin (apigenin 7-O-glucoside) can scavenge ROS in *Oryza sativa* [72] and it also showed a strong inhibition against free radical-induced oxidative damage and anti-inflammation on erythrocytes [73]. It has been reported in [74] that Apigenin 7-O-glucoside promoted cell apoptosis and inhibits cell migration in cervical cancer HeLa cells. Metabolites such as formononetin have also shown significant anticancer properties by regulating numerous signaling pathways to induce cell apoptosis against carcinogenic cells [75]. It has been showed in [76] that isoliquiritigenin can significantly enhance antitumor activity and inhibited the genotoxic effect of cyclophosphamide. Daidzein and genistein are considered as phyto-oestrogens which may protect against a wide range of conditions including breast, prostate, bowel and other forms of cancer, cardiovascular disease and, osteoporosis [77]. The significant number of phenolics increased by WL compared to ST is probably involved in ROS scavenging and plant cell wall protection via the lignin biosynthesis in *D. odorifera* leaflets. A comparative analysis between WL- and ST-treated seedlings showed that the ability of *D. odorifera* to respond more positively to waterlogging allowed a decrease in ROS accumulation which provided a better water status, cell homeostasis, photosynthesis, and shoot biomass accumulation in WL-treated seedlings compared to those in ST treatment. Indeed, WL and ST up-regulated PSMs compared to the control group. However, WL affected the PSMs significantly compared to ST-treated seedlings. Thus, the level of up-regulated PSMs is crucial in plant stress tolerance. Moreover, strong regulation of PSMs by WL showed the efficiency of the non-enzymic antioxidant in ROS scavenging and the promotion of therapeutic substances in medicinal plants.

## Conclusion

In conclusion, the results of the present study are very promising in the use of stress-induced to improve the capacity of medical trees to produce highly therapeutic substances. It has also highlighted the multiple roles of phenolics in plants under abiotic stresses as a powerful antioxidant tools and their possible implication in plant cell wall protection. Moreover, despite the aggressiveness of high ST in *D. odorifera* seedlings, WL-treated seedlings showed better phenolic accumulation in *D. odorifera* leaflets. However, under salinity important phenolics such as Apigenin and genistein were up-regulated. The results support the fact that a medical plant performance depend on it sensitivity to an environmental stress. Moreover a decrease of L-phenylalanine reflected a significant accumulation of aromatic compounds. The findings support investigating the specific roles of different phenolic compounds in response to abiotic stresses. Explore their antioxidative properties, protective roles in plant cell walls, and potential benefits for human health when these medicinal trees are used for herbal remedies. However, more studies focus on mechanistic studies to understand how environmental stressors, such as ST and WL, impact the biosynthesis of medicinal compounds, and the regulation of phenolics in plant tissues is needed. Researchers can also consider studying the economic and ecological implications of using stress-induced methods to enhance medicinal compound production in woody plants, which can help assess the feasibility and sustainability of such practices.

## Materials and methods

### Plant material and growth conditions

As described in a previous study, *D. odorifera* seedlings were obtained from Hainan Island (China) [78]; indeed, *D. odorifera* is endemic to Hainan. Saplings of *D. odorifera* were purchased from a wholesale plant nursery located in Ledong County (18°42′57.91′′N, 108°52′18.65′′E), Hainan Province, China. The seedlings were transplanted in pots (21 cm in diameter and 19 cm in height) and cut off 10 cm from the substratum surface for re-sprouting (60 days), thereafter healthy seedlings of the same size were chosen for different treatments application. Seedlings of *D. odorifera* were grown in plastic pots containing mixed soil in a greenhouse located at Hainan University, situated at coordinates 20° 03′ 22.80" North, 110° 19′ 10.20" East.. The substratum (about 4.5 kg each pot) comprised red soil, river sand, and coconut coir (2:1:1, v/v/v). The physicochemical properties of the red soil and the weather conditions surrounding the greenhouse were described by earlier research work [79] (Supplementary file 1). The present research comprised two experiments conducted from March to July: a preliminary experiment (30 days) and the main trial (6 days).

### Experimental setup

For both experiments, seedlings were distributed in a completely randomized design. About, 30 pots were then waterlogged. The water level was 10 cm above the soil surface and water evaporated was replaced to keep the water level during the experiment. Salinity (ST) was imposed by irrigating the plants with three different concentrations of NaCl solution (100 mM, 150 mM, and 200 mM) for the preliminary experiment (30 days) and one concentration of NaCl (200 mM) for the main trial (6 days). The salt treatment has been applied every three days during the experiment (100% field capacity). Seedlings were watered with clean water the day after salt treatment, to avoid excessive salt accumulation in the soil. Overall each group of treated seedlings was composed of 30 seedlings. The seedlings of the control group were watered every two days with clean water (100% field capacity). Healthy mature leaflets of *D. odorifera* were used for physiological and metabolomic analysis. The molecular, biochemical, and physiological measurements have been performed with samples harvested at early stress stages (day 6).

### Salinity damage index

The stress damage index (SDI) was used to determine the phenotypic responses of seedlings exposed to different treatments for the preliminary experiment. The method has been applied according to [80]. The SDI was based on the visual observation related to chlorosis and necrosis in the *D. odorifera* leaflet under CK, ST, or WL. The scoring system was between 0 and 10; leaflets presenting no visual symptoms were given a 0 score and 10 for the dead seedlings.

### Measurements of leaflet water status

The leaflet water content (LWC) was quantified as described by [81] with some modifications. Fresh leaflets were weighed (FW) and then dried at 80 °C for 48 h. The dried material was measured and recorded (DW). The leaflet water content was determined by the following formula: WC (%) = (FW—DW) / FW ∗ 100.

The leaflet relative turgidity (LRT), leaflet dry matter (LDM), and leaflet moisture (LM) were measured and calculated according to [82, 83] with some modifications. Fresh mature leaflets have been selected (4 replicates) and 6 discs were taken from each replicate. The disc leaflet weight (Fw) was measured, and then samples were placed into tubes filled with distilled water (10 mL) for 24 h. The mass of the turgid disc leaves was measured (Tw) and then placed into a dry machine at 80 °C for 24 h to obtain the dry weight (Dw).

$${\varvec{R}}{\varvec{T}} = 100 * [(Fw - Dw) / (Tw - Dw)]; {\varvec{D}}{\varvec{M}} = (Dw / Tw); {\varvec{L}}{\varvec{M}}\boldsymbol{ }=\boldsymbol{ }100 * [(Fw - Dw) / Dw]$$

### Measurements of leaflet membrane permeability

Leaflet relative conductivity (LRC) was determined as described in a previous research paper [78]. Meanwhile the Dew point water potential (DWP) was measured with a Dewpoint PotentiaMeter WP4 (Gene Company Ltd, USA).

### Measurements of leaflet anatomical traits

The leaf anatomical responses of *D. odorifera* seedlings were observed in 6 replicates for each treatment. The leaflet samples were fixed for 24 h in a 50% FAA fixative solution (Servicebio, Wuhan, China), and then the samples were sent to the Service Company (Servicebio Biomart Biotech Co., Ltd. Wuhan, China). The leaves anatomy was observed under orthostatic microscope (Nikon Eclipse E100, Nikon, JAPAN) and the images were taken for analysis using CaseViewer.

### Measurements of photosynthetic indexes

The chlorophylls and carotenoid contents were quantified with 80% of acetone (v/v) at 663, 646, and 470 nm, and the formula described by [84] was used to determine the concentrations. Gas exchange measurements including P_N_ (net photosynthetic rate), Trr (transpiration rate), Gs (stomatal conductance) and Wue (water use efficiency) were measured simultaneously with a portable photosynthesis system (LI-COR 6400, LI-COR Inc., USA). The third leaf from the top of the plants was selected for the experiments from 9: 00 am to 11: am while they were still attached to the plant.

### Measurements reactive oxygen species and lipid peroxidation contents

Hydrogen peroxide (H_2_O_2_) and hydroxyl free radical scavenging capacity (^●^OH) were measured with two assay kits Solarbio BC3590 (Beijing Solarbio Science and Technology Co., Ltd.) and Elisa kit (YT-F-KY013) respectively. The determination of H_2_O_2_ was based on its reaction with titanium sulfate that generated a yellow titanium peroxide complex and the absorbance was at 415 nm. The ^●^OH was measured based on Fenton reaction which is the most common reaction that generates hydroxyl free radical. Indeed, H_2_O_2_ is proportional to the amount of ^●^OH generated in Fenton reaction and the absorbance was read at 532 nm. The superoxide anions content (O_2_^●−^) was determined according to a modified colorimetric method from [85] and as described [79]. The principle of the reaction was based on the reaction with hydroxylamine hydrochloride, and then with p-aminobenzene sulfonic acid and α-naphthylamine. The mixture was kept at 25 °C for 20 min, and the absorbance read at 530 nm. The lipid peroxidation (MDA) content was determined using the thiobarbituric–trichloroacetitic acid (TBA–TCA) method. The colorimetric procedure of [86] was used to quantified the MDA, and the absorbance was read at 532, 600 and 450 nm. About 0.1 g of fresh leaf samples and 1 mL extraction solution (provided by assay kit for H_2_O_2_ and ^●^OH) were used during the determination of ROS and MDA. About 2.5 mL of 5% (w/v) TCA was used as extraction solution for MDA measurement and phosphate buffer (pH 7.8) for O_2_^●−^ determination.

### Antioxidant molecules, flavonoids and total of phenol measurements

Ascorbic acid (AsA) content was quantified via an assay kit (Solarbio, BC1230) at 265 nm based on the reaction between ascorbate oxidase and AsA to form dehydroascorbic acid, meanwhile assay kit (Solarbio, BC1170) was used for the reduced glutathione (GSH) content. GSH undergoes a reaction with 5,5'-dithiobis-(2-nitrobenzoic acid) (DTNB) to form 2-nitro-5-mercaptobenzoic acid and glutathione disulfide and the absorbance was read at 412 nm. The plant flavonoids were determined with a colorimetric assay kit (Solarbio, BC1330) at 470 nm, and the plant total of phenol was measured by the Folin-Ciocalteu method according to the manufacturer’s instructions at 760 nm (Solarbio, BC1340). ASA and GSH were extracted in 0.1 g of fresh leaf samples with 1 mL of extraction solution given by the assay kits. The total phenol was extracted with 2.5 mL of 60% alcohol and the flavonoids with 1 mL of 60% ethanol.

### Enzymes activities measurements

The antioxidant enzymes were measured spectrophotometrically with Solarbio assay kits according to the manufacturer’s instructions: Peroxidase (POD) activity (Solarbio, BC0090) at 470 nm, superoxide dismutase (SOD) activity (Solarbio, BC0170) at 560 nm, catalase (CAT) activity (Solarbio, BC0200) at 240 nm and at last glutathione peroxidase (GPX) activity (Solarbio, BC1190) at 412 nm. The POD activity was determined following its ability to catalyze H_2_O_2_, and the SOD activity was based on its ability to remove O_2_^●−^ and inhibit the formation of methionine. The lighter the blue color of the reaction solution, the higher the activity of SOD. The CAT activity was measured based on its ability to decompose H_2_O_2_ into the water and the presence of oxygen, and the GPX activity on its capacity to catalyze the oxidation of GSH by hydrogen peroxide to produce oxidized glutathione. About 0.1 g of fresh leaf samples were homogenized in 1 mL of extract solution (phosphate buffer solution) provided by the assay kits for POD, SOD, and CAT determination, and 0.05 g was used for GPX measurement.

The activities of other enzymes related to abiotic stresses were also quantified; these enzymes don’t take part to ROS scavenging directly compared to the antioxidant enzymes. Polyphenol oxidase (PPO) activity, phenylalanine ammonia-lyase (PAL) activity, plant lipoxygenase (LOX) activity were quantified with Solarbio assay kits: BC0190, BC0210 and BC0320 respectively. The absorbance was read at 410 nm (PPO), 290 nm (PAL) and 234 nm (LOX). The principle of the PPO activity determination was based on its capacity to catalyze o-dihydroxybenzene to produce quinones and the PAL activity on its ability to decompose L-phenylalanine into trans-cinnamic acid. The LOX activity measurement was based on its ability to catalyze the oxidation of linoleic acid. Fresh leaf samples (0.1 g) homogenized in 1 mL of extract solution provided by the assay kits were used for PPO, PAL, and LOX activity measurement. Plant alternative oxidase proteins (AOX) was evaluated with a quantitative sandwich ELISA kit (JL22749, Lot 05/2022, Plant AOX ELISA KIT; 48 T/96 T). The concentration of AOX was calculated by comparing the optical density of the samples to a standard curve at 450 nm. Fresh leaf samples were homogenized with phosphate buffer (10 mg of tissues to 100 µL). The reaction was based on the formation of a complex antibody–antigen–antibody–enzyme with an AOX antibody labeled.

### Metabolomic analysis

#### Sample extraction and HPLC conditions

The methodology described by [87] was used for the metabolomic analysis with some modifications. The samples were taken from 3 replicates for each treatments as mentioned by [88, 89]. About 100 mg of powder have been taken from freeze-dried leaf (Harvested at day 6) crushed with a mixer mill (MM 400, Retsch) with a zirconia bead for 1.5 min at 30 Hz. The extraction solution was obtained with 1.0 mL of 70% aqueous methanol and then centrifuged at 10, 000 g for 10 min. The extracts were absorbed (CNWBOND Carbon-GCB SPE Cartridge, 250 mg, 3 ml; ANPEL, Shanghai, China) and filtrated (SCAA-104, 0.22 μm pore size; ANPEL, Shanghai, China) before LC–MS analysis. The samples described above were analyzed by an LC–ESI–MS/MS system (HPLC, Shim-pack UFLC SHIMADZU CBM30A system, Hong Kong, China; MS, Applied Biosystems 6500 Q TRAP, Massachusetts, United States. The analytical parameters were as follow: HPLC column, Waters ACQUITY UPLC HSS T3 C18 (1.8 µm, 2.1 mm*100 mm); solvent system, water (0.04% acetic acid): acetonitrile (0.04% acetic acid); gradient program, 95:5 V/V at 0 min, 5:95 V/V at 11.0 min, 5:95 V/V at 12.0 min, 95:5 V/V at 12.1 min, 95:5 V/V at 15.0 min; flow rate, 0.40 ml/min; temperature, 40 °C; injection volume: 2 μl. The effluent was alternatively joined to an ESI-triple quadrupole-linear ion trap (Q TRAP)-MS.

### ESI-Q TRAP-MS/MS

Triple quadrupole-linear ion trap mass spectrometer (Q TRAP), API 6500 Q TRAP LC/MS/MS System was employed toward Linear Ion Trap (LIT) and triple quadrupole (QQQ) scans. The system was connected with an ESI Turbo Ion-Spray interface, functioning in a positive ion mode and controlled by Analyst 1.6.3 software (AB Sciex). The electrospray ionization (ESI) source operation parameters, instrument tuning, and mass calibration were literally as described by [87]. The collision gas (N2) was arranged at 5 psi during QQQ scans based on Multiple Reaction Monitoring (MRM) analysis. The de-clustering potential (DP) and collision energy (CE) for individual MRM transitions was performed with further DP and CE optimization. For each period according to the metabolites eluted within this period, a specific set of MRM transitions were monitored.

### Quantitative and qualitative principles of metabolites

The secondary spectral data obtained were qualitatively analyzed based on public metabolite database (MassBank, KNApSAcK, Metlin, MoTo DB and hmdb) and a self-built database MetWare database (MWDB). The isotope signal and the repetitive signal of K^+^, Na^+^ and, NH_4_^+^ were removed during the analysis. The metabolites were quantified via MRM of triple quadrupole mass spectrometry as described by [90]. Moreover, the mass spectrometry total ion chromatogram of mixed samples, MRM metabolite detection multimodal plot, metabolite quantitative analysis integration calibration chart and the QC sample mass detection TIC overlay.

### Statistical data analysis

Mass spectral data were processed using the software Analyst 1.6.3. Multivariate statistical analysis methods, including principal component analysis (PCA) and orthogonal partial least squares discriminant analysis (OPLS-DA), were performed based on the method describe by [91]. The data from metabolite analysis were normalized and R software (www.r-project.org/) was used to perform cluster analysis (Hierarchical cluster analysis, HCA). The annotation of differential metabolites and metabolite enrichment pathway analysis were performed with Kyoto Encyclopedia of Genes and Genomes (KEGG) database (http://www.genome.- ad.jp/kegg/). The physiological and morphological data were statistically analyzed with one-way ANOVA and Tukey’s honestly significant difference test (Graph Pad prism 9.0.0). Data were expressed as means ± SD (at least 3 replicates), and significant differences between means were determined at a *p*-value ≤ 0.05.

### Supplementary Information

**Additional file 1.**

**Additional file 2.**

**Additional file 3.**

**Additional file 4.**

**Additional file 5.**

## Data Availability

All data generated or analyzed during this study are included in this published article as supplementary excel files: file 1 (morphological and physiological raw data) and file 2 (metabolomic raw data).

## References

[1] Geissman TA (1964). New substances of plant origin. Annu Rev Pharmacol.

[2] Austen N, Walker HJ, Lake JA, Phoenix GK, Cameron DD (2019). The regulation of plant secondary metabolism in response to abiotic stress: Interactions between heat shock and elevated CO_2_. Front Plant Sci.

[3] Ganjewala D, Kaur G, Srivastava N (2019). Metabolic engineering of stress protectant secondary metabolites to confer abiotic stress tolerance in plants.

[4] Saidi A (2022). Phenolic characterization using cLC-DAD analysis and evaluation of in vitro and in vivo pharmacological activities of *Ruta tuberculata* Forssk. Antioxidants.

[5] Niazian M, Howyzeh MS, Sadat-Noori SA (2021). Integrative effects of stress-and stress tolerance-inducing elicitors on in vitro bioactive compounds of ajowan [Trachyspermum ammi (L.) Sprague]. Plant Cell Tissue Organ Culture.

[6] Yeshi K, Crayn D, Ritmejerytė E, Wangchuk P (2022). Plant secondary metabolites produced in response to abiotic stresses has potential application in pharmaceutical product development. Molecules.

[7] Tavares WR, Seca AM, Inula L (2019). secondary metabolites against oxidative stress-related human diseases. Antioxidants.

[8] Hong SY, Roze L, Linz J (2013). Oxidative stress-related transcription factors in the regulation of secondary metabolism. Toxins.

[9] Demidchik V. Reactive oxygen species, oxidative stress and plant ion channels. In: Demidchik V, Maathuis F. (eds) Ion channels and plant stress responses. Signaling and communication in plants. Springer, Berlin, Heidelberg. 2010. 10.1007/978-3-642-10494-7_11

[10] Demidchik V (2015). Mechanisms of oxidative stress in plants: From classical chemistry to cell biology. Environ Exp Bot.

[11] Waszczak C, Carmody M, Kangasjärvi J (2018). Reactive oxygen species in plant signaling. Annu Rev Plant Biol.

[12] Rentel MC, Knight MR (2004). Oxidative stress-induced calcium signaling in *Arabidopsis*. Plant Physiol.

[13] Parsons HT, Fry SC (2010). Reactive oxygen species-induced release of intracellular ascorbate in plant cell-suspension cultures and evidence for pulsing of net release rate. New Phytol.

[14] Sharma A (2019). Phytohormones regulate accumulation of osmolytes under abiotic stress. Biomolecules.

[15] Bailey-Serres J, Mittler R (2006). The roles of reactive oxygen species in plant cells”. Plant Physiol.

[16] Dumanović J, Nepovimova E, Natić M, Kuča K, Jaćević V (2021). The significance of reactive oxygen species and antioxidant defense system in plants: A concise overview”. Front Plant Sci.

[17] Waśkiewicz A, Muzolf-Panek M, Goliński P. Phenolic content changes in plants under salt stress, in Ecophysiology and responses of plants under salt Stress, New York, NY: Springer New York, 2013, pp. 283–314.

[18] Guedes LM, Torres S, Sáez-Carillo K, Becerra J, Pérez CI, Aguilera N (2022). High antioxidant activity of phenolic compounds dampens oxidative stress in Espinosa nothofagi galls induced on Nothofagus obliqua buds. Plant Sci.

[19] El-Shora H (2002). Properties of phenylalanine ammonia-lyase from marrow cotyledons. Plant Sci.

[20] Wei J (2022). AetSRG1 contributes to the inhibition of wheat Cd accumulation by stabilizing phenylalanine ammonia lyase. J Hazard Mater.

[21] Araji S (2014). Novel roles for the polyphenol oxidase enzyme in secondary metabolism and the regulation of cell death in Walnut. Plant Physiol.

[22] Boeckx T, Webster R, Winters AL, Webb KJ, Gay A, Kingston-Smith AH (2015). Polyphenol oxidase-mediated protection against oxidative stress is not associated with enhanced photosynthetic efficiency. Ann Bot.

[23] Hasanuzzaman M (2020). Reactive oxygen species and antioxidant defense in plants under abiotic stress: Revisiting the crucial role of a universal defense regulator”. Antioxidants.

[24] Lim CW, Han SW, Hwang IS, Kim DS, Hwang BK, Lee SC (2015). The pepper lipoxygenase CaLOX1 plays a role in osmotic, drought and high salinity stress response. Plant Cell Physiol.

[25] Viswanath KK, Varakumar P, Pamuru RR, Basha SJ, Mehta S, Rao AD (2020). Plant lipoxygenases and their role in plant physiology. J Plant Biol.

[26] Singh P, Arif Y, Miszczuk E, Bajguz A, Hayat S (2022). Specific roles of lipoxygenases in development and responses to stress in plants. Plants.

[27] Ismail AM, Horie T (2017). Genomics, physiology, and molecular breeding approaches for improving salt tolerance. Annu Rev Plant Biol.

[28] Hernández JA (2019). Salinity tolerance in plants: Trends and perspectives. Int J Mol Sci.

[29] Etherington JR (1984). Comparative studies of plant growth and distribution in relation to waterlogging: X Differential formation of adventitious roots and their experimental excision in Epilobium hirsutum and Chamerion angustifolium. J Ecol.

[30] Qi X (2020). Sugar enhances waterlogging-induced adventitious root formation in cucumber by promoting auxin transport and signalling. Plant Cell Environ.

[31] Pan J, Sharif R, Xu X, Chen X (2021). Mechanisms of waterlogging tolerance in plants: Research progress and prospects. Front Plant Sci.

[32] Pang J, Cuin T, Shabala L, Zhou M, Mendham N, Shabala S (2007). Effect of secondary metabolites associated with anaerobic soil conditions on ion fluxes and electrophysiology in Barley roots. Plant Physiol.

[33] Shabala S (2011). Physiological and cellular aspects of phytotoxicity tolerance in plants: the role of membrane transporters and implications for crop breeding for waterlogging tolerance. New Phytol.

[34] Pandey S (2022). Biotechnological studies of medicinal plants to enhance production of secondary metabolites under environmental pollution, in Environmental pollution and medicinal plants.

[35] Briskin DP (2000). Medicinal plants and phytomedicines Linking plant biochemistry and physiology to human health. Plant Physiol.

[36] Zhao X, Wang C, Meng H, Yu Z, Yang M, Wei J (2020). *Dalbergia odorifera*: A review of its traditional uses, phytochemistry, pharmacology, and quality control. J Ethnopharmacol.

[37] Zhang DY, Zu YG, Fu YJ, Luo M, Gu CB, Wang W, Yao XY (2011). Negative pressure cavitation extraction and antioxidant activity of biochanin A and genistein from the leaves of Dalbergia odorifera T. Chen Sep Purif Technol.

[38] Ma L, Rao X, Chen X (2019). Waterlogging tolerance of 57 plant species grown hydroponically. HortScience.

[39] Rahman S, Iqbal M, Husen A. Medicinal Plants and Abiotic Stress: An Overview, in *Medicinal Plants*, Singapore: Springer Nature Singapore, 2023, pp. 1–34.

[40] Khan M (2000). Effects of salinity on growth, water relations and ion accumulation of the subtropical perennial halophyte. Atriplex griffithii var stocksii Ann Bot.

[41] Abdul Qados AMS. Effect of salt stress on plant growth and metabolism of bean plant Vicia faba (L.) Agric Sci 2011;10:7–15 10.1016/j.jssas.2010.06.002.

[42] Alam MA, Juraimi AS, Rafii MY, Abdul Hamid A. Effect of salinity on biomass yield and physiological and stem-root anatomical characteristics of purslane ( *Portulaca oleracea* L.) Accessions. Biomed Res. Int. 2015;2015:1–15 10.1155/2015/105695. 10.1155/2015/105695 PMC4352753 25802833

[43] Sack L, Holbrook NM (2006). Leaf hydraulics. Annu Rev Plant Biol.

[44] Buckley TN, John GP, Scoffoni C, Sack L (2015). How Does leaf anatomy influence water transport outside the xylem?. Plant Physiol.

[45] Jain P (2021). A minimally disruptive method for measuring water potential in planta using hydrogel nanoreporters. Proc Natl Acad Sci.

[46] Bartlett MK, Scoffoni C, Sack L (2012). The determinants of leaf turgor loss point and prediction of drought tolerance of species and biomes: a global meta-analysis. Ecol Lett.

[47] Ye H (2018). A major natural genetic variation associated with root system architecture and plasticity improves waterlogging tolerance and yield in soybean. Plant Cell Environ.

[48] Pagán E, Robles JM, Temnani A, Berríos P, Botía P, Pérez-Pastor A (2022). Effects of water deficit and salinity stress on late mandarin trees. Sci Total Environ.

[49] Hacke UG, Sperry JS (2001). Functional and ecological xylem anatomy. Perspect Plant Ecol Evol Syst.

[50] Mazur R (2021). The SnRK2.10 kinase mitigates the adverse effects of salinity by protecting photosynthetic machinery. Plant Physiol.

[51] Mittler R (2017). ROS are good. Trends Plant Sci.

[52] Pazmino DM, Rodriguez-Serrano M, Romero-Puertas MC, Archilla-Ruiz A, Del Rio LA, Sandalio LM (2011). Differential response of young and adult leaves to herbicide 2,4-dichlorophenoxyacetic acid in pea plants: role of reactive oxygen species. Plant Cell Environ.

[53] Sies H, Berndt C, Jones DP (2017). Oxidative Stress. Annu Rev Biochem.

[54] Kerchev PI, Van Breusegem F (2022). Improving oxidative stress resilience in plants. Plant J.

[55] Prasad A, Sedlářová M, Kale RS, Pospíšil P (2017). Lipoxygenase in singlet oxygen generation as a response to wounding: in vivo imaging in *Arabidopsis thaliana*. Sci Rep.

[56] Hernández I, Alegre L, Van Breusegem F, Munné-Bosch S (2009). How relevant are flavonoids as antioxidants in plants?. Trends Plant Sci.

[57] Vishwakarma A, Tetali SD, Selinski J, Scheibe R, Padmasree K (2015). Importance of the alternative oxidase (AOX) pathway in regulating cellular redox and ROS homeostasis to optimize photosynthesis during restriction of the cytochrome oxidase pathway in *Arabidopsis thaliana*. Ann Bot.

[58] Bartoli CG (2006). Inter-relationships between light and respiration in the control of ascorbic acid synthesis and accumulation in *Arabidopsis thaliana* leaves. J Exp Bot.

[59] Prasch CM, Sonnewald U (2013). Simultaneous application of heat, drought, and virus to *Arabidopsis* plants reveals significant shifts in signaling networks. Plant Physiol.

[60] Sarri E (2021). Salinity stress alters the secondary metabolic profile of *M. sativa*, *M. arborea* and their hybrid (Alborea). Int J Mol Sci.

[61] Chen Y, Huang W, Zhang F, Luo X, Hu B, Xie J (2021). Metabolomic profiling of Dongxiang wild rice under salinity demonstrates the significant role of amino acids in rice salt stress. Front Plant Sci.

[62] Kessler A, Kalske A (2018). Plant secondary metabolite diversity and species interactions”. Annu Rev Ecol Evol Syst.

[63] Hartmann T (2007). From waste products to ecochemicals: Fifty years research of plant secondary metabolism. Phytochemistry.

[64] Kanehisa M (2000). KEGG: Kyoto Encyclopedia of Genes and Genomes. Nucleic Acids Res.

[65] Zhang X, Liu CJ (2015). Multifaceted regulations of gateway enzyme phenylalanine ammonia-lyase in the biosynthesis of phenylpropanoids. Mol Plant.

[66] Zhang Y (2015). Multi-level engineering facilitates the production of phenylpropanoid compounds in tomato. Nat Commun.

[67] Barros J (2019). 4-Coumarate 3-hydroxylase in the lignin biosynthesis pathway is a cytosolic ascorbate peroxidase. Nat Commun.

[68] Rui Y, Dinneny JR (2020). A wall with integrity: surveillance and maintenance of the plant cell wall under stress. New Phytol.

[69] Li Y, Kim JI, Pysh L, Chapple C (2015). Four isoforms of *Arabidopsis thaliana* 4-coumarate: CoA ligase (4CL) have overlapping yet distinct roles in phenylpropanoid metabolism. Plant Physiol.

[70] Bernards MA, Lewis NG (1992). Alkyl ferulates in wound healing potato tubers. Phytochemistry.

[71] Al Maruf A, Lip H, Wong H, O’Brien PJ (2015). Protective effects of ferulic acid and related polyphenols against glyoxal- or methylglyoxal-induced cytotoxicity and oxidative stress in isolated rat hepatocytes. Chem Biol Interact.

[72] Zhan X, Shen Q, Chen J, Yang P, Wang X, Hong Y. Rice sulfoquinovosyltransferase SQD2.1 mediates flavonoid glycosylation and enhances tolerance to osmotic stress. Plant. Cell Environ. 2019;42:2215–2230. 10.1111/pce.13554. 2019, 10.1111/pce.13554. 10.1111/pce.13554 30942482

[73] Wang W (2020). Efficiency comparison of apigenin-7-O-glucoside and trolox in antioxidative stress and anti-inflammatory properties. J Pharm Pharmacol.

[74] Liu MM (2020). Apigenin 7-O-glucoside promotes cell apoptosis through the PTEN/PI3K/AKT pathway and inhibits cell migration in cervical cancer HeLa cells. Food Chem Toxicol.

[75] Tay KC (2019). Formononetin: A review of its anticancer potentials and mechanisms. Front Pharmacol.

[76] Zhao H (2013). “Isoliquiritigen enhances the antitumour activity and decreases the genotoxic effect of Cyclophosphamide. Molecules.

[77] Liggins J, Bluck LJC, Runswick S, Atkinson C, Coward WA, Bingham SA (2000). Daidzein and genistein contents of vegetables. Br J Nutr.

[78] Cisse EHM, Miao LF, Yang F, Huang JF, Li DD, Zhang J (2021). Gly betaine surpasses melatonin to improve salt tolerance in *Dalbergia*
*odorifera*. Front Plant Sci.

[79] Li DD, Cisse EHM, Guo L, Zhang J, Miao L, Yang F (2022). Comparable and adaptable strategies to waterlogging stress regulated by adventitious roots between two contrasting species. Tree Physiol.

[80] Falakboland Z, Zhou M, Zeng F, Kiani-Pouya A, Shabala L, Shabala S (2017). Plant ionic relation and whole-plant physiological responses to waterlogging, salinity and their combination in barley. Funct Plant Biol.

[81] Jin X, Shi C, Yu CY, Yamada T, Sacks EJ (2017). Determination of leaf water content by visible and near-infrared spectrometry and multivariate calibration in *Miscanthus*. Front Plant Sci.

[82] Barrs H, Weatherley P (1962). A re-examination of the relative turgidity technique for estimating water deficits in leaves. Aust J Biol Sci.

[83] Saura-Mas S, Lloret F (2007). Leaf and shoot water content and leaf dry matter content of mediterranean woody species with different post-fire regenerative strategies. Ann Bot.

[84] Lichtenthaler HK, Wellburn AR (1983). “Determinations of total carotenoids and chlorophylls a and b of leaf extracts in different solvents. Biochem Soc Trans.

[85] Zhang S, Lu S, Xu X, Korpelainen H, Li C (2010). Changes in antioxidant enzyme activities and isozyme profiles in leaves of male and female *Populus cathayana* infected with *Melampsora larici-populina*. Tree Physiol.

[86] Yang F, Xu X, Xiao X, Li C (2009). Responses to drought stress in two poplar species originating from different altitudes. Biol Plant.

[87] Chen W (2013). A novel integrated method for large-scale detection, identification, and quantification of widely targeted metabolites: Application in the study of rice metabolomics. Mol Plant.

[88] Watson BS (2015). “Integrated metabolomics and transcriptomics reveal enhanced specialized metabolism in *Medicago truncatula* root border cells. Plant Physiol.

[89] Zhu Y (2021). Combined transcriptomic and metabolomic analysis reveals the role of phenylpropanoid biosynthesis pathway in the salt tolerance process of *Sophora alopecuroides*. Int J Mol Sci.

[90] Li Q, Song J (2019). Analysis of widely targeted metabolites of the euhalophyte *Suaeda salsa* under saline conditions provides new insights into salt tolerance and nutritional value in halophytic species. BMC Plant Biol.

[91] Eriksson L, Andersson PL, Johansson E, Tysklind M (2006). Megavariate analysis of environmental QSAR data. Part I – A basic framework founded on principal component analysis (PCA), partial least squares (PLS), and statistical molecular design (SMD). Mol Divers.

