# Supplementary material for: Physio-biochemical and metabolomic responses of the woody plant Dalbergia odorifera to salinity and waterlogging
Source: BMC Plant Biol. 2024 Jan 13;24:49. doi: 10.1186/s12870-024-04721-5 (PMC10787392; doi:10.1186/s12870-024-04721-5)
Supplement: Supplementary file 3 — Additional file 3. [file 12870_2024_4721_MOESM3_ESM.docx]

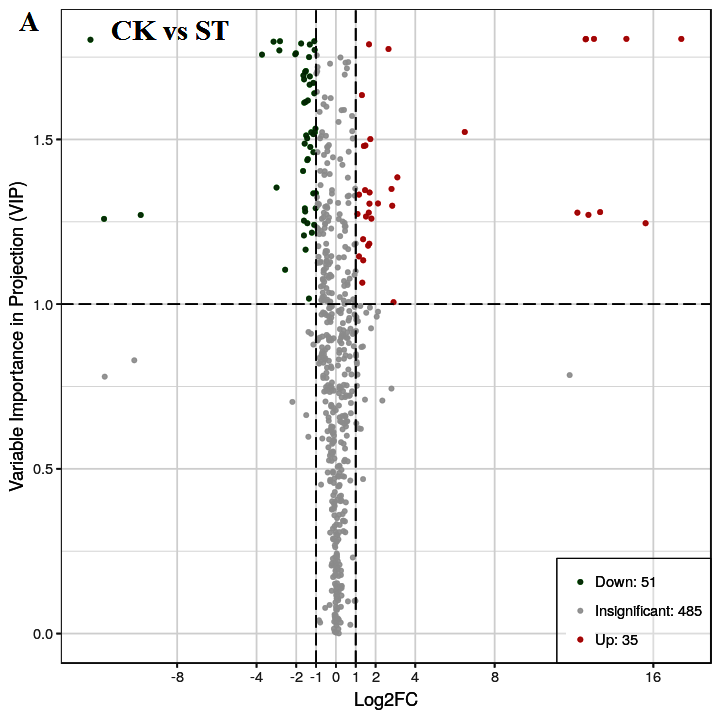


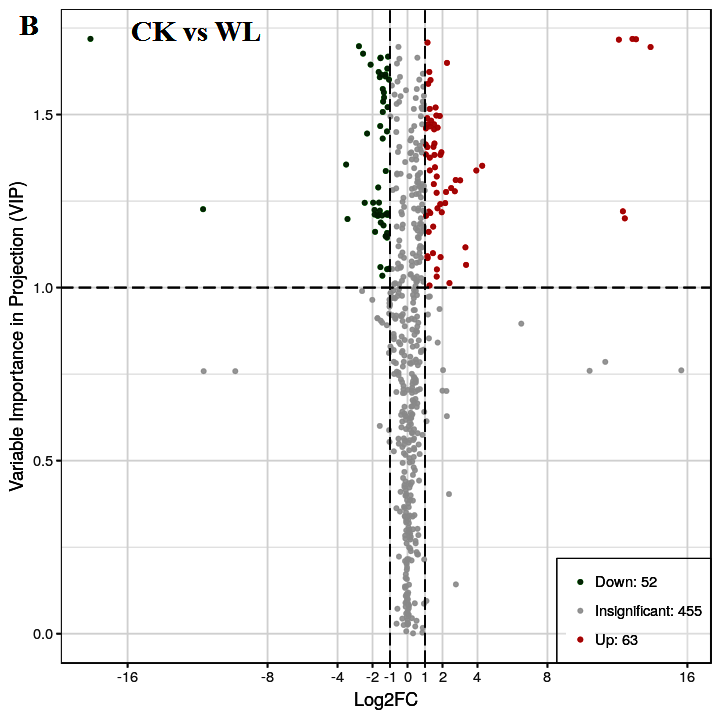


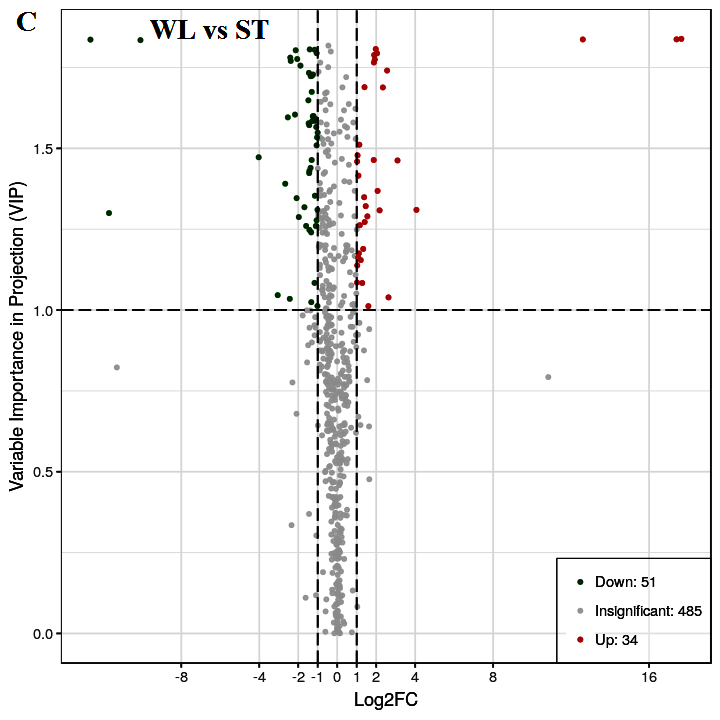

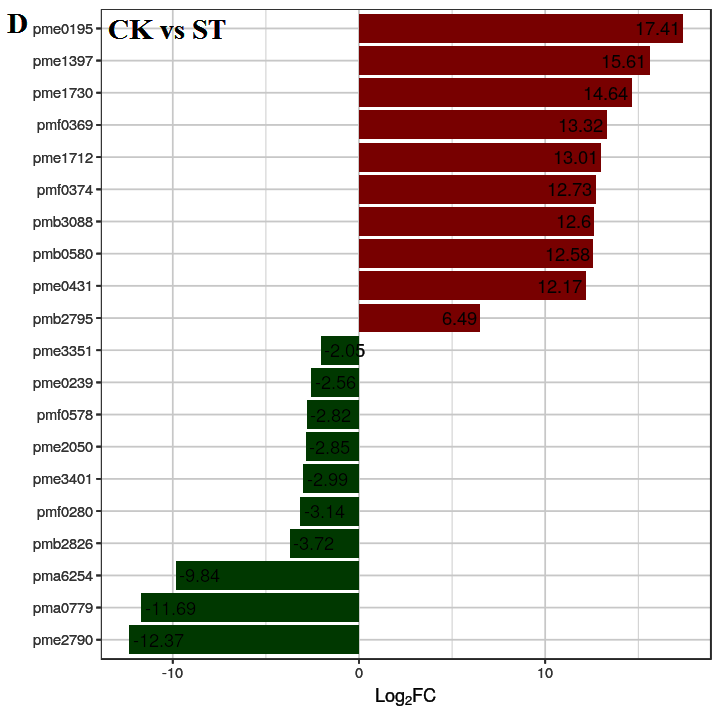

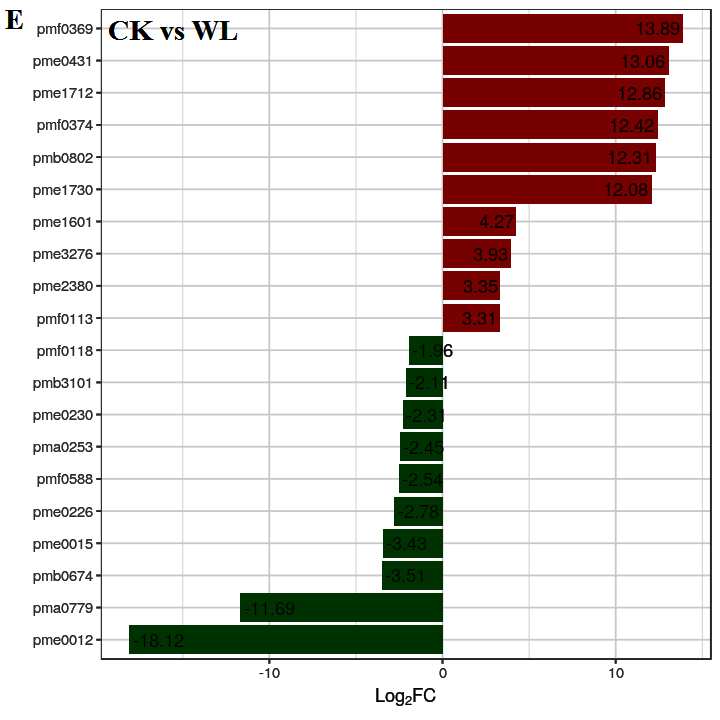


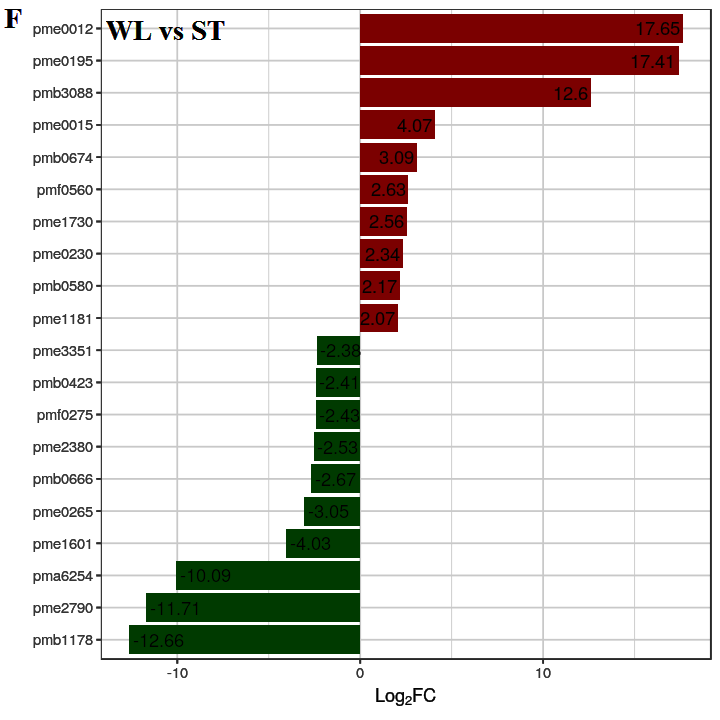


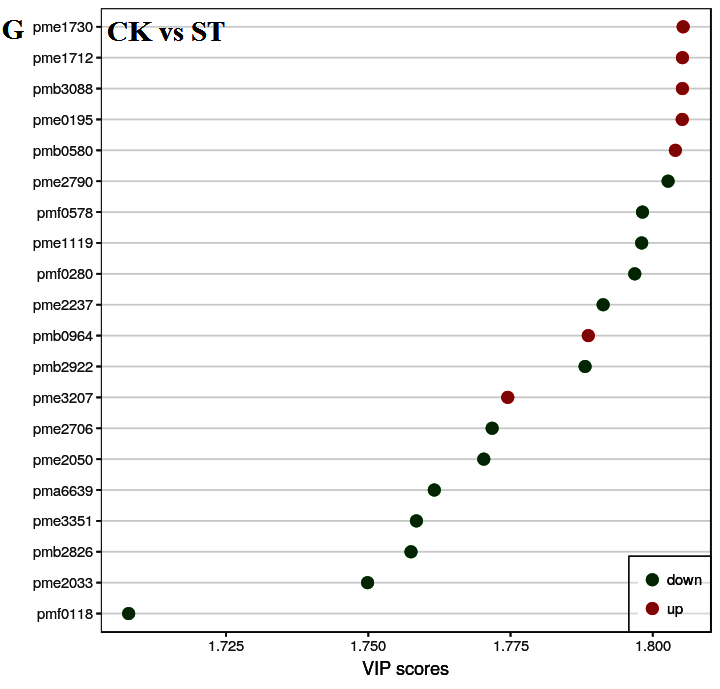


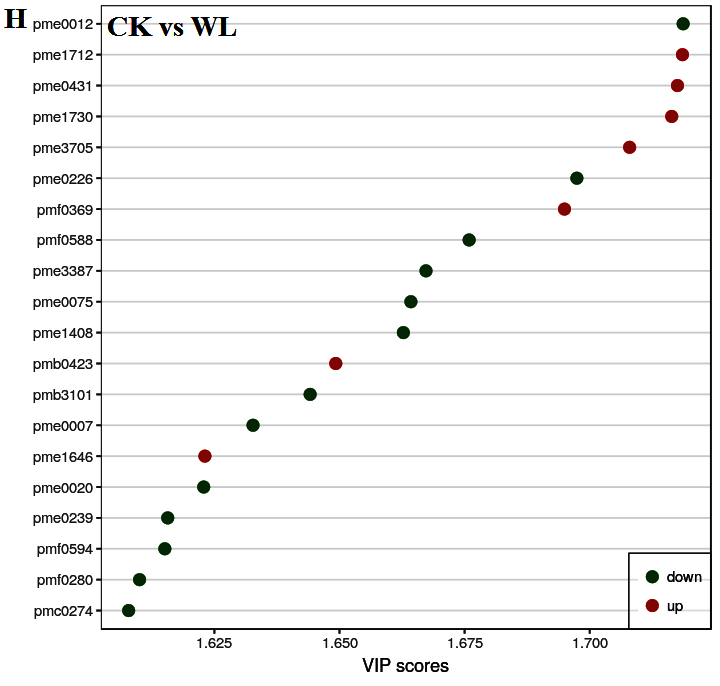


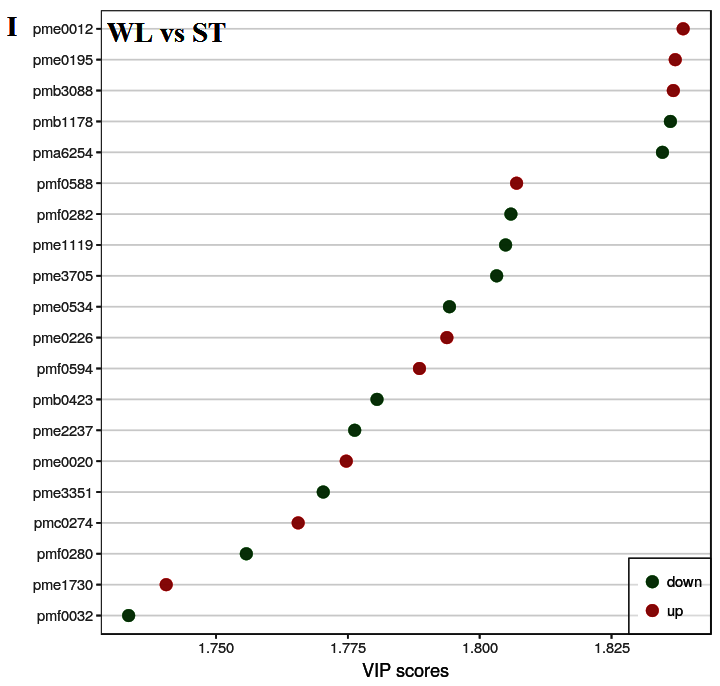


**Suppl. Fig. 1** Variable importance in project (VIP, A-C), multiples histogram representing the differentially expressed metabolites with log2 fold changes (D-F), differential metabolite VIP value (G-I) in *Dalbergia odorifera* leaflets under control (CK) vs ST (first column), CK vs WL (second column) and ST vs WL (third column)


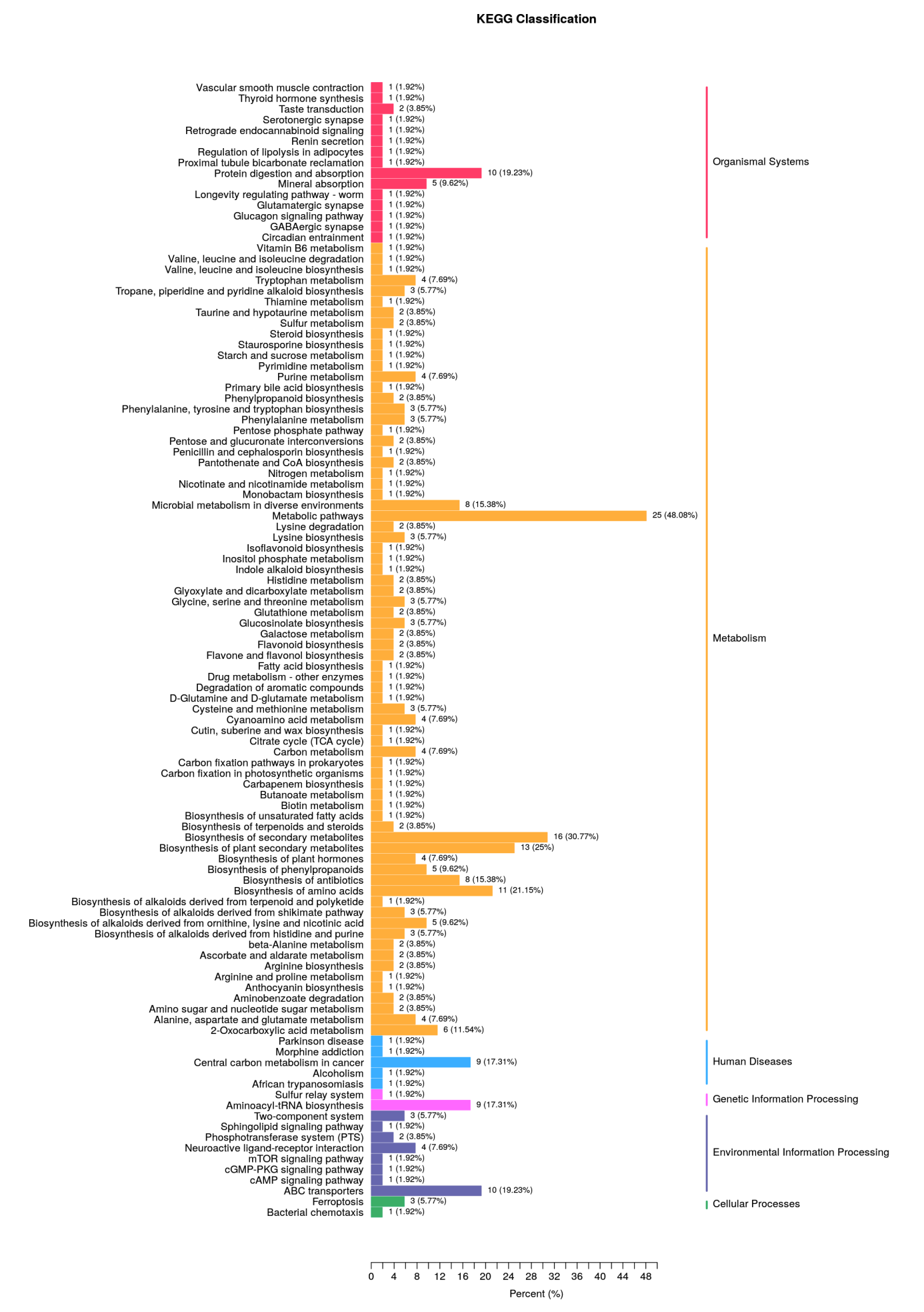


**Suppl. Fig. 2** Classification of the differential metabolite KEGG enrichment maps in *D. odorifera* leaflets under waterlogging (WL) vs salinity (ST)


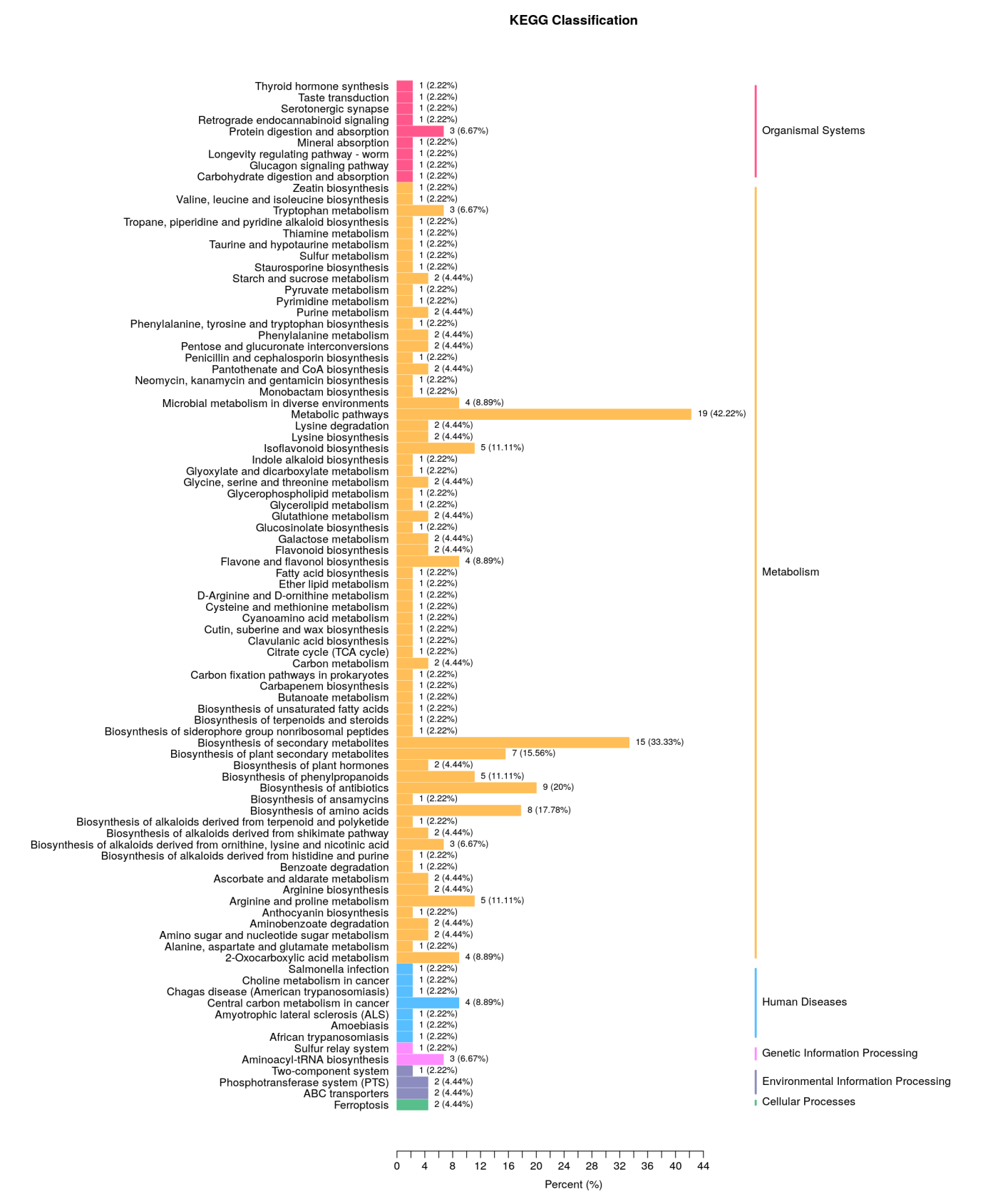


**Suppl. Fig. 3** Classification of the differential metabolite KEGG enrichment maps in *D. odorifera* leaflets under control (CK) versus salinity (ST).


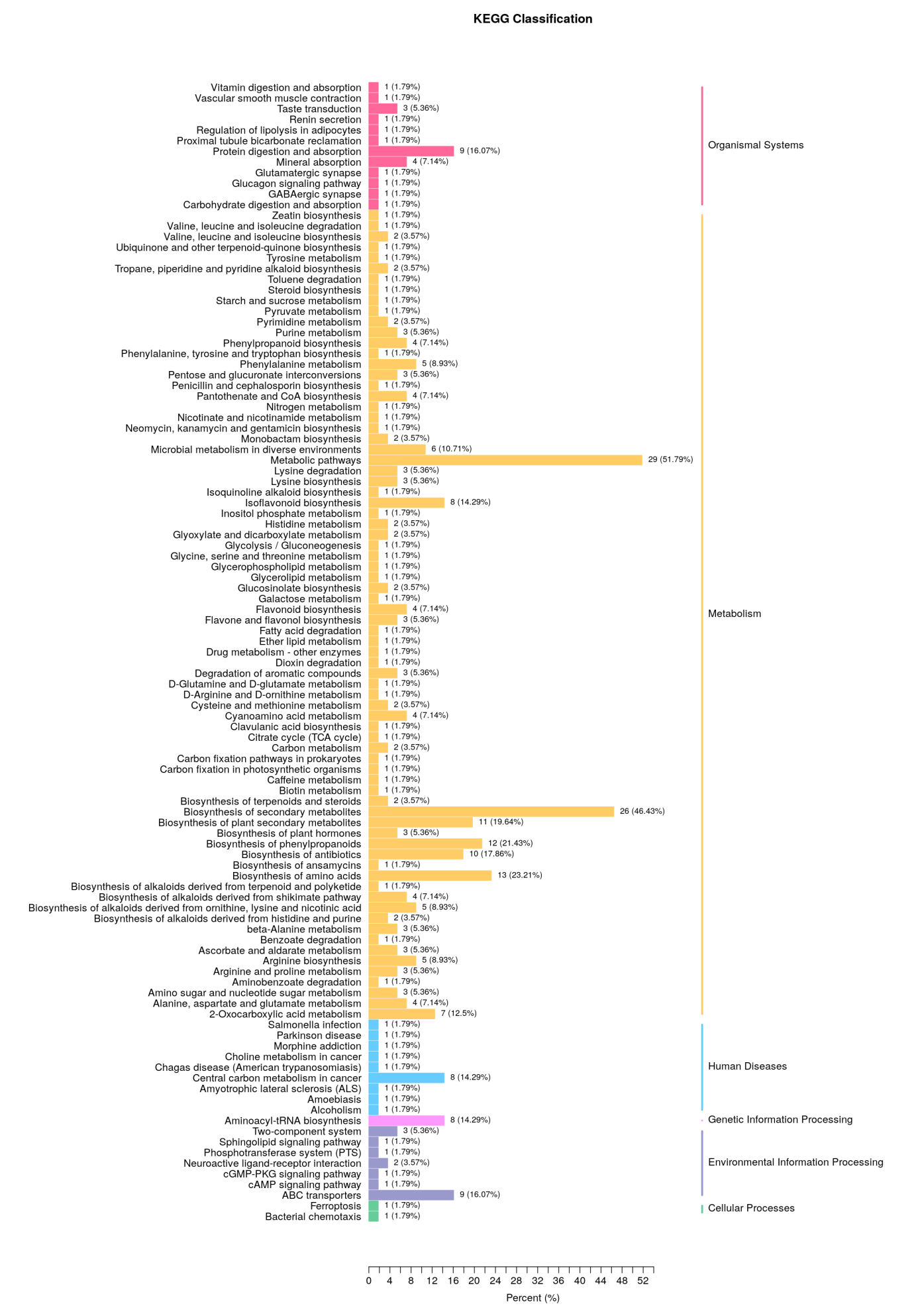
**Suppl. Fig. 4** Classification of the differential metabolite KEGG enrichment maps in *D. odorifera* leaflets under control (CK) versus waterlogging (WL).


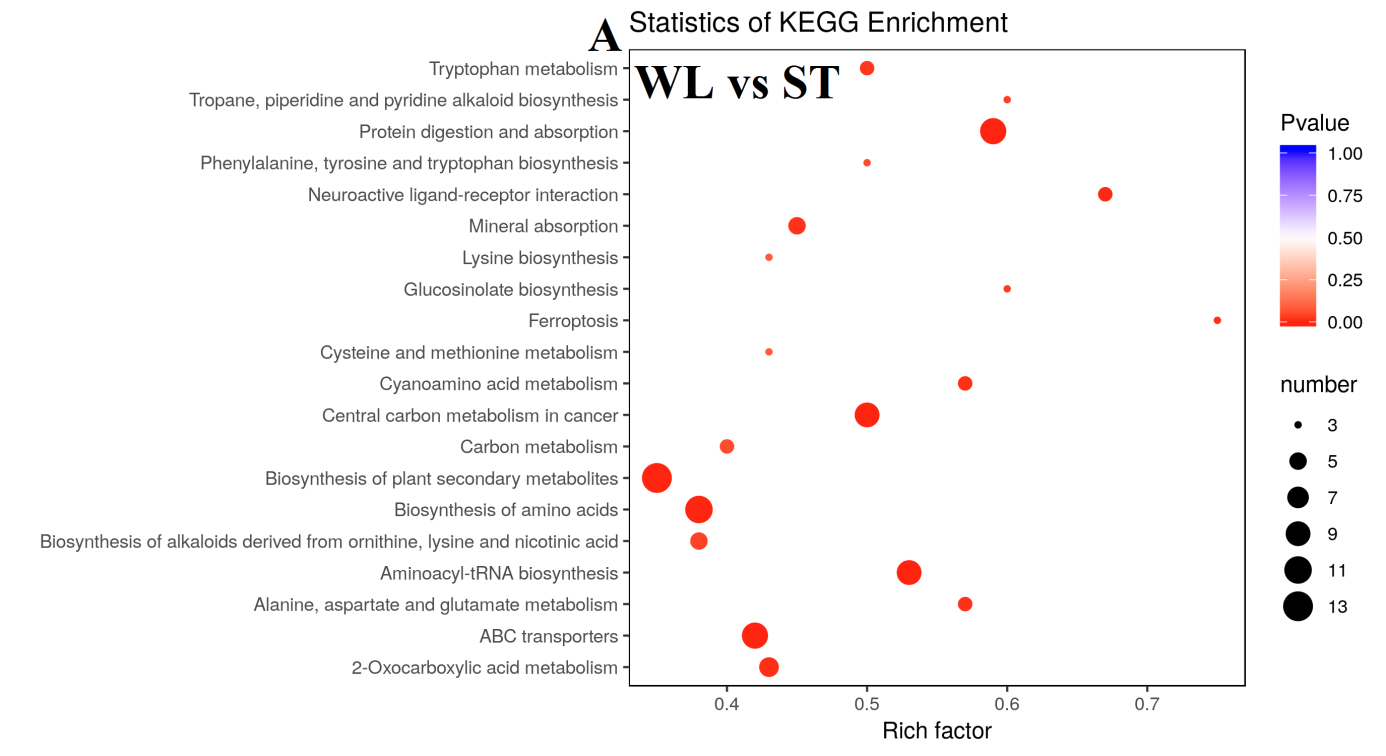


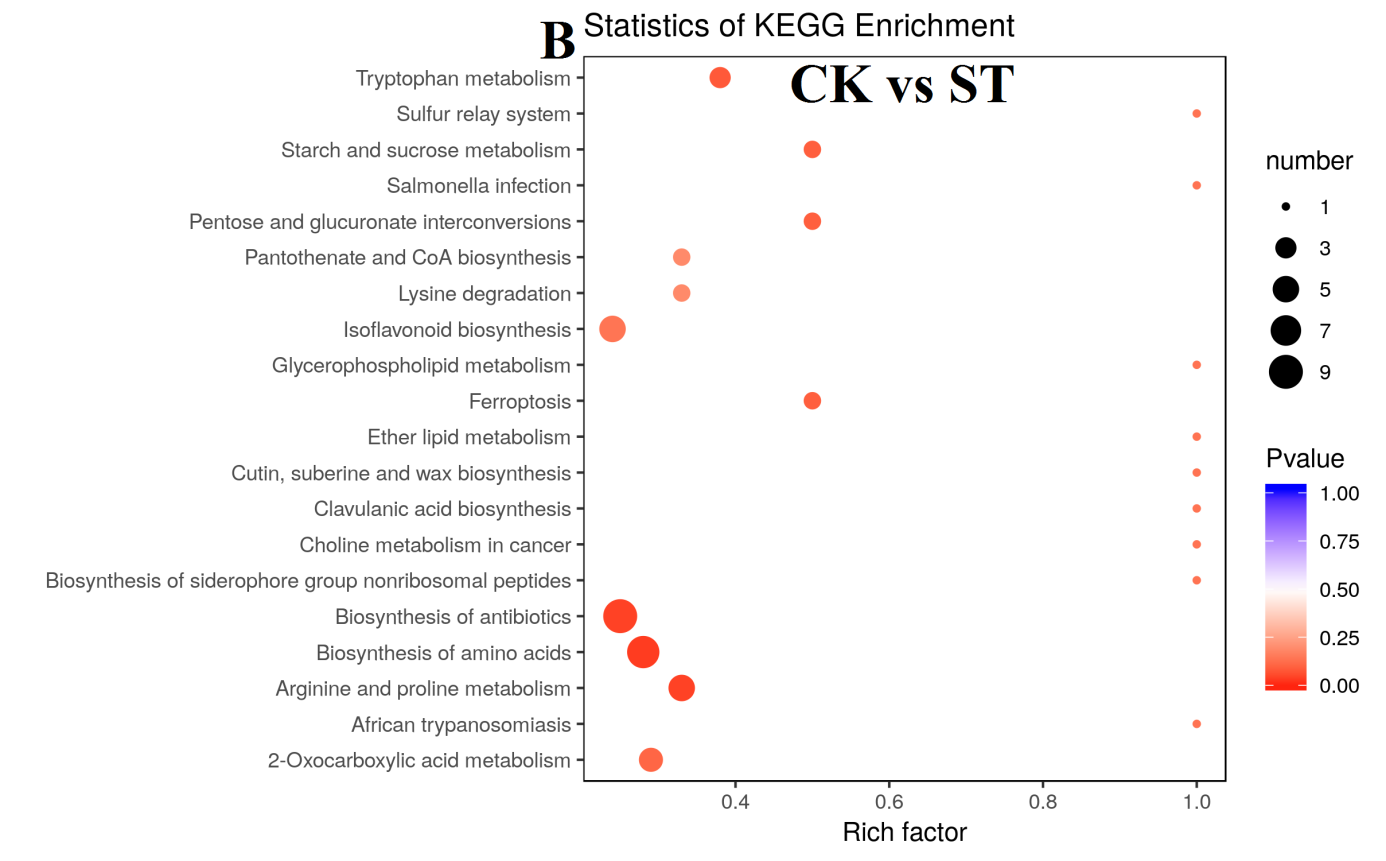


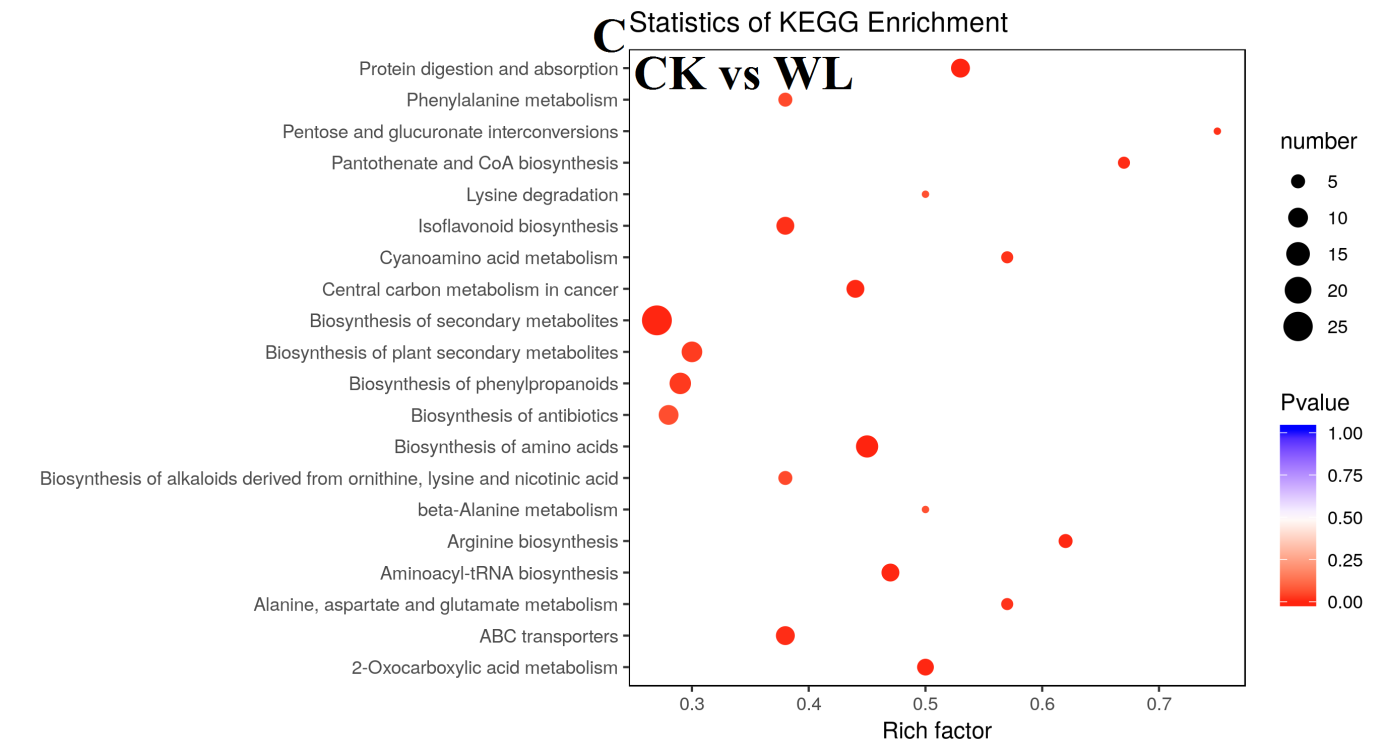


**Suppl. Fig. 5** statistics of the differential metabolite KEGG enrichment maps in *D. odorifera* leaflets under control (CK) vs salinity (ST) (A), CK vs waterlogging (WL) (B) and WL vs ST (C). The redder corresponded to a significant enrichment. The size of the dots represents the number of enriched differential metabolites.


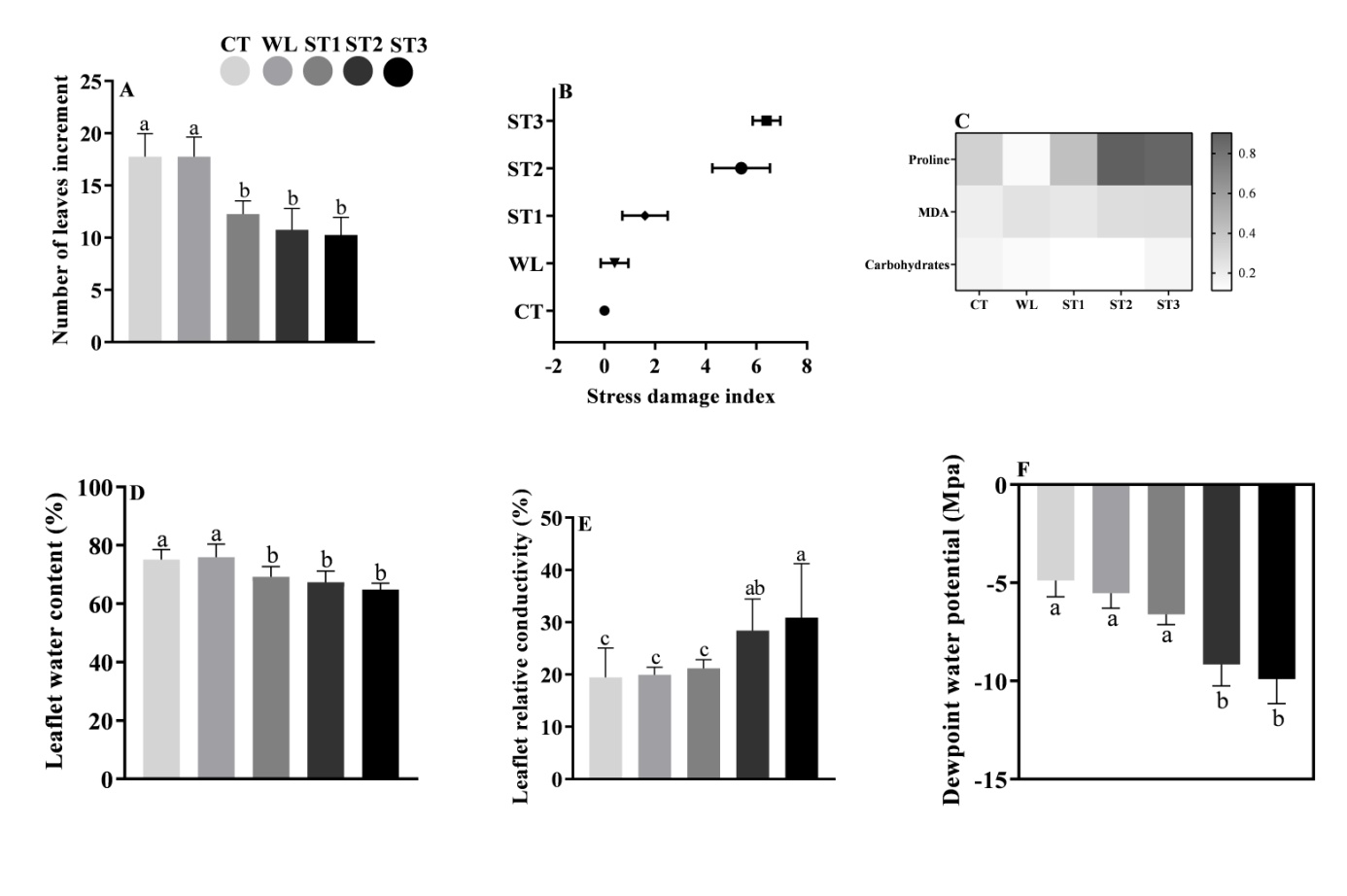
**Suppl. Fig. 6** Variation in number of leaves increment (A) stress damage index (B) proline, MDA and carbohydrates contents (C), leaflet water content (D), leaflet relative conductivity (D) and dew-point water potential (E) in *D. odorifera* leaflets under waterlogging and different level of salinity (100 mM, 150 mM and 200 mM).


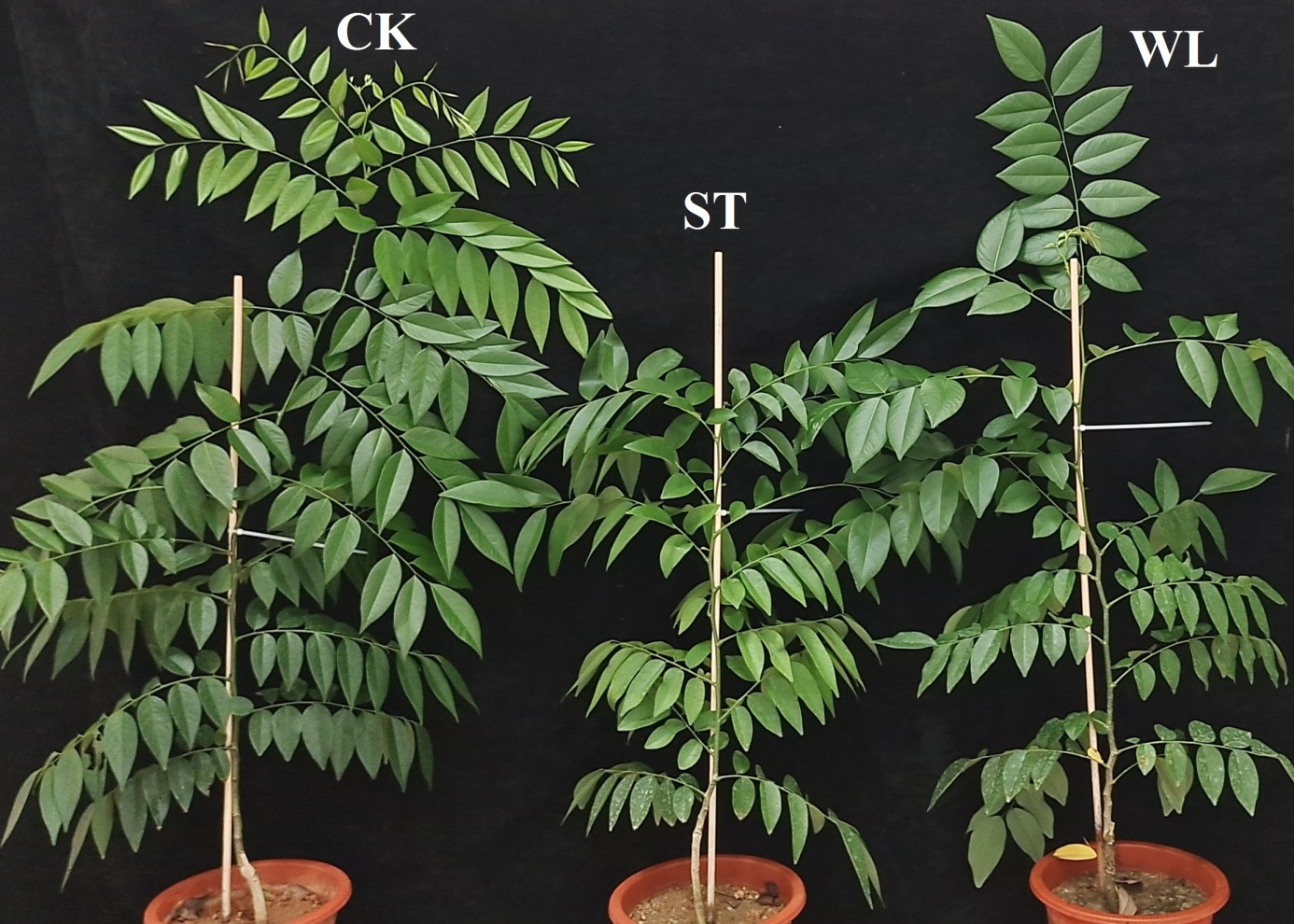


**Suppl. Fig. 7** Pictures of *D. odorifera* under salinity and waterlogging at day 6. Treatments are presented as follows: control (CK), salinity (ST), and waterlogging (WL).
