# Supplementary material for: Physio-biochemical and metabolomic responses of the woody plant Dalbergia odorifera to salinity and waterlogging
Source: BMC Plant Biol. 2024 Jan 13;24:49. doi: 10.1186/s12870-024-04721-5 (PMC10787392; doi:10.1186/s12870-024-04721-5)
Supplement: Supplementary file 4 — Additional file 4. [file 12870_2024_4721_MOESM4_ESM.docx]

**Physicochemical properties of the soil and the conditions surrounding the greenhouse**

The substratum was composed of red soil, were collected from the experimental site. They contained 5.34 mg.kg⁻¹ of ammoniacal nitrogen, 11.78 mg.kg⁻¹ of available phosphorus, and 81.72 mg.kg⁻¹ of available potassium. All pots containing saplings were placed in a greenhouse at Hainan University (20°03′22.80′′N, 110°19′10.20′′E), where ambient rainfall was blocked, but normal light and temperature conditions were maintained. This region experiences a typical tropical monsoon climate characterized by high temperatures and rainfall. The annual average for rainfall, temperature, and sunlight exposure in this area is 1715.3 mm, 24.4°C, and 2000 hours, respectively (source: Meteorological Bureau of Haikou, http://www.hkqx.net).
