# Supplementary material for: Physio-biochemical and metabolomic responses of the woody plant Dalbergia odorifera to salinity and waterlogging
Source: BMC Plant Biol. 2024 Jan 13;24:49. doi: 10.1186/s12870-024-04721-5 (PMC10787392; doi:10.1186/s12870-024-04721-5)
Supplement: Supplementary file 5 — Additional file 5. [file 12870_2024_4721_MOESM5_ESM.docx]

Table S1: Stem height and leaf area increment and, the survival rate of *Dalbergia odorifera* under salinity and waterlogging on the 30th day.

| Indexes | Treatments | | | | |
| --- | --- | --- | --- | --- | --- |
|  | **Control** | **Salinity (100 mM)** | **Salinity (150 mM)** | **Salinity (200 mM)** | **Waterlogging** |
| Stem Height increment (cm) | 31.80 ± 1.09 a | 26.21 ± 2.31 c | 22.15 ± 1.58 d | 20.40 ± 0.96 d | 29.55 ± 0.67 b |
| Leaf area (cm^2^) | 22.25 ± 1.27 a | 18.26 ± 0.64 b | 16.32 ± 2.33 c | 16.05 ± 1.21 c | 21.94 ± 0.94 a |
| Survival rate (%) | 100 | 82 | 58 | 53 | 100 |

Note: Stem height and leaf area are expressed as mean ± standard deviation, different lowercase letters indicate the significant difference after multiple comparisons test between different treatments at P<0.05.

Table S2: Regulation status of the main metabolites involved in phenolic coumarins biosynthesis pathway in *Dalbergia odorifera* leaflets under 200 mM of salinity (ST) or waterlogging (WL)

| **Phenolic coumaryl pathway** | |
| --- | --- |
| **Under ST** | **Under WL** |
| Aesculetin (**unchanged)** | Aesculetin (**unchanged**) |
| Scopoletin (**unchanged**) | Scopoletin (**unchanged**) |
| Scopolin (**unchanged**) | Scopolin (**unchanged**) |
| 7-hydroxycoumarin (**unchanged**) | 7-hydroxycoumarin (**unchanged**) |

Table S3: Regulation status of the main metabolites involved in phenylpropanoids biosynthesis pathway in *Dalbergia odorifera* leaflets under 200 mM of salinity (ST) or waterlogging (WL)

| **Phenylpropanoid biosynthesis pathway** | |
| --- | --- |
| **Under ST** | **Under WL** |
| p-Coumarate (**unchanged**) | p-Coumarate (**unchanged**) |
| p-Coumaryl alcohol (**unchanged**) | p-Coumarate (**up-regulated**) |
| Caffeate (**unchanged**) | Caffeate (**up-regulated**) |
| Ferulate (**unchanged**) | Ferulate (**up-regulated**) |
| Coniferyl alcohol (**unchanged**) | Coniferyl alcohol (**unchanged**) |
| Coniferyl aldehyde (**unchanged**) | Coniferyl aldehyde (**unchanged**) |
| Sinapate (**unchanged**) | Sinapate (**unchanged**) |
| Sinapyl alcohol (**unchanged**) | Sinapyl alcohol (**unchanged**) |

Table S4: Regulation status of the main metabolites involved in flavonoids biosynthesis pathway in *Dalbergia odorifera* leaflets under 200 mM of salinity (ST) or waterlogging (WL)

| **Flavonoid biosynthesis pathway** | |
| --- | --- |
| **Under ST** | **Under WL** |
| Pinocembrin (**unchanged**) | Pinocembrin (**unchanged**) |
| Isoliquiritigenin (**unchanged**) | Isoliquiritigenin (**up-regulated**) |
| Liquiritigenin (**unchanged**) | Liquiritigenin (**up-regulated**) |
| 7,4-Dihydroxy- flavone (**unchanged**) | 7,4-Dihydroxy- flavone (**unchanged**) |
| Butein (**unchanged**) | Butein (**up-regulated**) |
| Butin (**unchanged**) | Butin (**unchanged**) |
| Phlorizin (**unchanged**) | Phlorizin (**unchanged**) |
| Prunin (**unchanged**) | Prunin (**unchanged**) |
| Naringin (**unchanged**) | Naringin (**unchanged**) |
| Hesperetin (**unchanged**) | Hesperetin (**unchanged**) |
| Naringenin (**unchanged**) | Naringenin (**unchanged**) |
| Naringenin chalcone (**unchanged**) | Naringenin chalcone (**unchanged**) |
| Dihydrokaempferol (**unchanged**) | Dihydrokaempferol (**unchanged**) |
| Galangin (**unchanged**) | Galangin (**unchanged**) |
| Dihydrokaempferol (**unchanged**) | Dihydrokaempferol (**unchanged**) |
| Pelargonidin (**up-regulated**) | Pelargonidin (**unchanged**) |
| Epiafzelechin (**unchanged**) | Epiafzelechin (**unchanged**) |
| Kaempferol (**unchanged**) | Kaempferol (**unchanged**) |
| Apigenin (**up-regulated**) | Apigenin (**up-regulated**) |
| Cyanidin (**unchanged**) | Cyanidin (**unchanged**) |
| Eriodictyol (**unchanged**) | Eriodictyol (**unchanged**) |
| Luteolin (**unchanged**) | Luteolin (**unchanged**) |
| Homoeriodictyol (**unchanged**) | Homoeriodictyol (**unchanged**) |
| Caffeoyl quinic acid (**unchanged**) | Caffeoyl quinic acid (**unchanged**) |
| Biochanin A (**unchanged**) | Biochanin A (**unchanged**) |
| Genistein (**up-regulated**) | Genistein (**up-regulated**) |
| Formononetin (**unchanged**) | Formononetin (**up-regulated**) |
| Daidzein (**unchanged**) | Daidzein (**up-regulated**) |

Table S5: Regulation status of the main metabolites involved in flavone and flavonol biosynthesis pathway in *Dalbergia odorifera* leaflets under 200 mM of salinity (ST) or waterlogging (WL)

| **Flavone and flavonol biosynthesis pathway** | |
| --- | --- |
| **Under ST** | **Under WL** |
| Apigenin **(up-regulated)** | Apigenin **(up-regulated)** |
| Cosmosin **(unchanged)** | Cosmosin (**unchanged**) |
| Apiin **(unchanged)** | Apiin (**unchanged**) |
| Rhoifolin **(unchanged)** | Rhoifolin (**unchanged**) |
| Luteoloside **(unchanged)** | Luteoloside (**unchanged**) |
| Luteolin **(unchanged)** | Luteolin (**unchanged**) |
| Syringetin **(down-regulated)** | Syringetin **(down-regulated)** |
| Rutin **(up-regulated)** | Rutin **(up-regulated)** |
| Isoquercitrin **(unchanged)** | Isoquercitrin (**unchanged**) |
| Ayarin **(unchanged)** | Ayarin **(unchanged)** |
| Trifolin **(unchanged)** | Trifolin **(unchanged)** |
| Astragalin **(unchanged)** | Astragalin **(unchanged)** |
| Kaempferide **(unchanged)** | Kaempferide **(unchanged)** |
| Kaempferol **(unchanged)** | Kaempferol **(unchanged)** |
| Kaempferin **(unchanged)** | Kaempferin **(unchanged)** |
| 3,7-O-dimethylquercetin **(down-regulated)** | 3,7-O-dimethylquercetin **(unchanged)** |

Table S6: Regulation status of the main metabolites involved in isoflavonoid biosynthesis pathway in *Dalbergia odorifera* leaflets under 200 mM of salinity (ST) or waterlogging (WL)

| **Isoflavonoid biosynthesis pathway** | |
| --- | --- |
| **Under ST** | **Under WL** |
| 2'-Hydroxydaidzein **(unchanged)** | 2'-Hydroxydaidzein **(unchanged)** |
| 7'4-Dihydroxy-flavone **(unchanged)** | 7'4-Dihydroxy-flavone **(unchanged)** |
| Formononetin 7-0-glucoside **(unchanged)** | Formononetin 7-0-glucoside **(unchanged)** |
| Liquiritigenin **(unchanged)** | Liquiritigenin **(up-regulated)** |
| Daidzein **(unchanged)** | Daidzein **(up-regulated)** |
| Formononetin **(unchanged)** | Formononetin **(up-regulated)** |
| Calycosin **(unchanged)** | Calycosin **(unchanged)** |
| 6'-Hydroxydaidzein **(up-regulated)** | 6'-Hydroxydaidzein **(unchanged)** |
| Glycitein **(up-regulated)** | Glycitein **(unchanged)** |
| 2'-Hydoxygenistein **(up-regulated)** | 2'-Hydoxygenistein **(up-regulated)** |
| Apigenin **(up-regulated)** | Apigenin **(up-regulated)** |
| Prunetin **(unchanged)** | Prunetin **(unchanged)** |
| Naringenine **(unchanged)** | Naringenine **(unchanged)** |
| Biochanin A **(unchanged)** | Biochanin A **(unchanged)** |
| Biochanin A7-O-glucoside **(unchanged)** | Biochanin A7-O-glucoside **(unchanged)** |
| Genistein **(up-regulated)** | Genistein **(up-regulated)** |
| Genistein7-O-glucoside **(unchanged)** | Genistein7-O-glucoside **(unchanged)** |
| Vestitol **(unchanged)** | Vestitol **(up-regulated)** |
| Medicarpin **(unchanged)** | Medicarpin **(unchanged)** |
| Maackiain **(unchanged)** | Maackiain **(up-regulated)** |
